# Towards designer polyolefins: highly tuneable olefin copolymerisation using a single permethylindenyl post-metallocene catalyst†

**DOI:** 10.1039/d3sc04861f

**Published:** 2023-12-06

**Authors:** Clement G. Collins Rice, Louis J. Morris, Jean-Charles Buffet, Zoë R. Turner, Dermot O'Hare

**Affiliations:** a Chemistry Research Laboratory, Department of Chemistry, University of Oxford 12 Mansfield Road Oxford OX1 3TA UK dermot.ohare@chem.ox.ac.uk

## Abstract

Using a highly active permethylindenyl-phenoxy (PHENI*) titanium catalyst, high to ultra-high molecular weight ethylene–linear-α-olefin (E/LAO) copolymers are prepared in high yields under mild conditions (2 bar, 30–90 °C). Controllable, efficient, and predictable comonomer enchainment provides access to a continuum of copolymer compositions and a vast range of material properties using a single monomer-agnostic catalyst. Multivariate statistical tools are employed that combine the tuneability of this system with the analytical and predictive power of data-derived models, this enables the targeting of polyolefins with designer properties directly through predictive alteration of reaction conditions.

## Abstract 2

Using a highly active permethylindenyl-phenoxy (PHENI*) titanium catalyst, high to ultra-high molecular weight ethylene–linear-α-olefin copolymers are prepared in high yields, with exceptional comonomer affinity, and predictable, tuneable composition.

## Graphical abstract

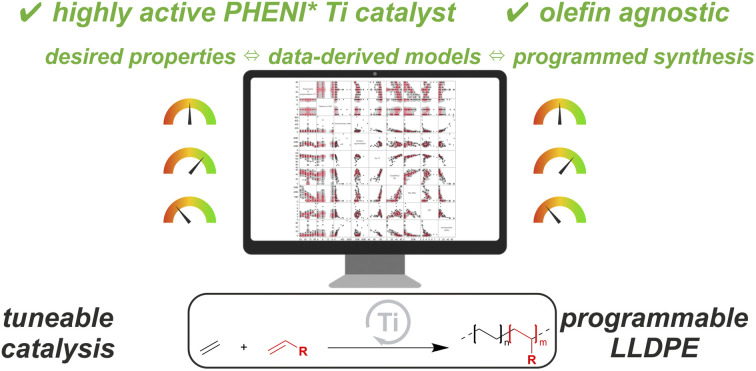

## Introduction

The incorporation of linear α-olefins (LAOs) into ethylene (E) polymers allows for control over short-chain branching, enabling tuneability of the chemical and physical properties of the resulting linear low density polyethylene (LLDPE) polymers.^1–5^ Propylene (C3) and higher olefins up to at least 1-hexacosene (C26) have been studied as comonomers for ethylene co-polymerisation.^6–14^ LLDPE accounts for around a quarter of total polyethylene demand, and principally finds applications in films, packaging, sealants, plasticisers, modifiers, and compatibilisers.^15–21^ The structure and properties of linear low density polyethylenes have been the subject of intense study, with the degree and distribution of short-chain branching having a strong influence on morphological, thermal, and mechanical properties.^22,23^ It has, for example, been shown that polymer melting temperatures decrease linearly as a function of branching density.^24–30^ Commercial LLDPEs are typically random copolymers, with the distribution of monomers determined statistically, and can be produced with unimodal or bimodal interchain comonomer composition distributions.^31^ The length of the side chains has proven significant in determining the physical properties of LLDPEs, with impact strength, ductility, and impact fatigue life increasing with increasing branch length.^32^

Olefin polymerisation systems are typically high-complexity multicomponent systems. Unpredictable and nonlinear relationships exist between reaction conditions and the end composition and properties of the polymer. Furthermore, enchainment of ethylene is normally greatly preferred over LAOs.^33,34^ Thus, the targeted synthesis of an LLDPE material with pre-defined properties is non-trivial and reliant upon extensive screening and empirical optimisation of catalyst and reaction conditions. Klosin *et al.* recently noted that no single catalyst is able to fulfil the requirements of the increasingly broad range of polyolefin products, and highlighted the desirability of “programmable and predictable polymerisation performance”.^35^ A single catalyst that is efficiently able to copolymerise a range of olefins to high molecular weight polymers with readily and predictably tuneable composition is elusive, remaining a highly desirable industrial goal.

Traditional heterogeneous Ziegler–Natta catalysts have been used in the copolymerisation of ethylene with LAOs with high activities but relatively limited comonomer incorporation.^36,37^ Metallocenes have shown good homogeneous catalytic activity towards E/LAO copolymerisation when activated with methylaluminoxane (MAO), with a more uniform comonomer distribution than is possible with heterogeneous systems.^38–40^

With the development of post-metallocene catalysts such as the Constrained Geometry Complexes (CGCs),^41^ it was shown that ligands with electron-donating substituents had increased activities and afforded copolymers with increased molecular weights and high comonomer incorporations, up to 25 mol% C8.^35,42^ Quijada *et al.* showed that *ansa*-bridged metallocenes enabled higher LAO incorporation than unbridged analogues.^40^ Group four Phenoxy-Induced Complexes of Sumitomo (PHENICS) utilising Cp-,^43,44^ Ind-,^45^ and Flu-derived^46^ apical ligands have demonstrated LAO incorporations of up to 35 mol%, reported with the complex {(η^5^-Cp)Me_2_C(PhO)}TiCl_2_.^44^ Compared to CGCs, PHENICS complexes were found to result in greater E/C6 copolymerisation activities as well as high comonomer incorporation.^44,45^

Irwin *et al.* reported the “sterically expanded” complex ^Me_2_^SB(Oct,^^*t*^Bu^N)ZrCl_2_·OEt_2_ ({(η^1^-C_29_H_36_)Me_2_Si(^*t*^BuN)}ZrCl_2_·OEt), based on a monohapto octamethyloctahydrodibenzofluorenyl (Oct) ligand, which is the only example of a catalyst to demonstrate activities and C8 incorporations proportional to comonomer concentration.^47^ Initial activities as high as 81 000 kg_LLDPE_ mol_Zr_^−1^ h^−1^ bar^−1^ and incorporations of 75 mol% were reported for reactions conducted in neat 1-octene (6400 mM), for 50 seconds under 5.5 bar ethylene pressure at 75 °C. In this case an “unyielding comonomer effect” describes the phenomenon of increasing activities with comonomer concentration, and is attributed to the dominant electronic advantages of substituted α-olefins for an electron rich “sterically indiscriminate” complex.^48^ While this result is remarkable, the steric expansion also rendered termination processes facile, resulting in very low polymer molecular weights (*M*_w_ < 7 kDa),^49^ severely limiting the industrial utility of this catalyst. Varying the Oct ligand substituents was able to increase *M*_w_ but at the expense of activity and control, with *Đ* > 100 reported.^49^ Furthermore, the ease with which this complex reinserts olefinic macromonomers leads to long chain branching, reducing the predictability of the system.^50,51^

Single-site catalysts of sufficiently high activity towards LAO enchainment may allow access to a largely diffusion-limited monomer-agnostic regime, where copolymer composition is simply determined by the relative concentrations of comonomer. Recently, we reported solid-supported group four permethylindenyl-phenoxy (PHENI*) catalysts, which display outstanding performance for ethylene and propylene polymerisation, producing ultrahigh molecular weight homopolymers,^52,53^ and tuneable copolymers.^54^ In the latter case, ethylene–propylene copolymers were produced with polymer composition almost exactly corresponding to the feed ratio of gaseous monomers.

**Fig. 1:**
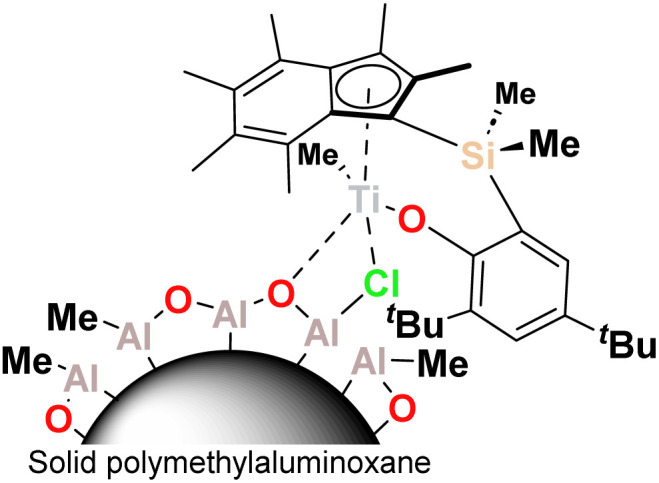
Schematic structure of sMAO-supported PHENI* catalyst 1.

In this work we describe the application of the PHENI* complex ^Me_2_^SB(^^*t*^Bu_2_^ArO,I*)TiCl_2_ ({(η^5^-C_9_Me_6_)Me_2_Si(2,4-^*t*^Bu_2_-C_6_H_2_O)}TiCl_2_), supported on solid MAO (1; Fig. 1), for the copolymerisation of ethylene with LAOs. Virtually unprecedented levels of single-catalyst tuneability allowed statistical modelling to be applied as a predictive tool in the targeted synthesis of designer polyolefins.

## Results and discussion

The sMAO-supported PHENI* complex sMAO–^Me_2_^SB(^^*t*^Bu_2_^ArO,I*)TiCl_2_ ([Al_sMAO_]_0_/[Ti]_0_ = 200; 1) was prepared according to literature procedures,^52,53^ and utilised in slurry-phase olefinic copolymerisations. Heterogenised single-site catalysts can be used in gas-phase and slurry-phase processes to overcome morphological constrains and reactor fouling typical of homogeneous systems. Organometallic complexes for olefin copolymerisations have been successfully immobilised on a range of solid supports including silica,^55^ solid polymethylaluminoxane (sMAO),^56^ layered double hydroxide (LDH),^57,58^ and (aminomethyl)polystyrene.^59^ Polymerisations were carried out within a temperature of polymerisation (*T*_p_) range of 30 ≤ *T*_p_ ≤ 90 °C under 2 bar ethylene pressure. 10 mg of 1 (∼712 nmol Ti) and the cocatalyst TIBA ([Al_TIBA_]_0_/[Ti]_0_ = 1000) were utilised with 50 mL hexanes as a diluent. Liquid comonomers were added to the reaction mixture simultaneously with the introduction of the gaseous monomer.

### Ethylene/LAO copolymerisation

**Fig. 2:**
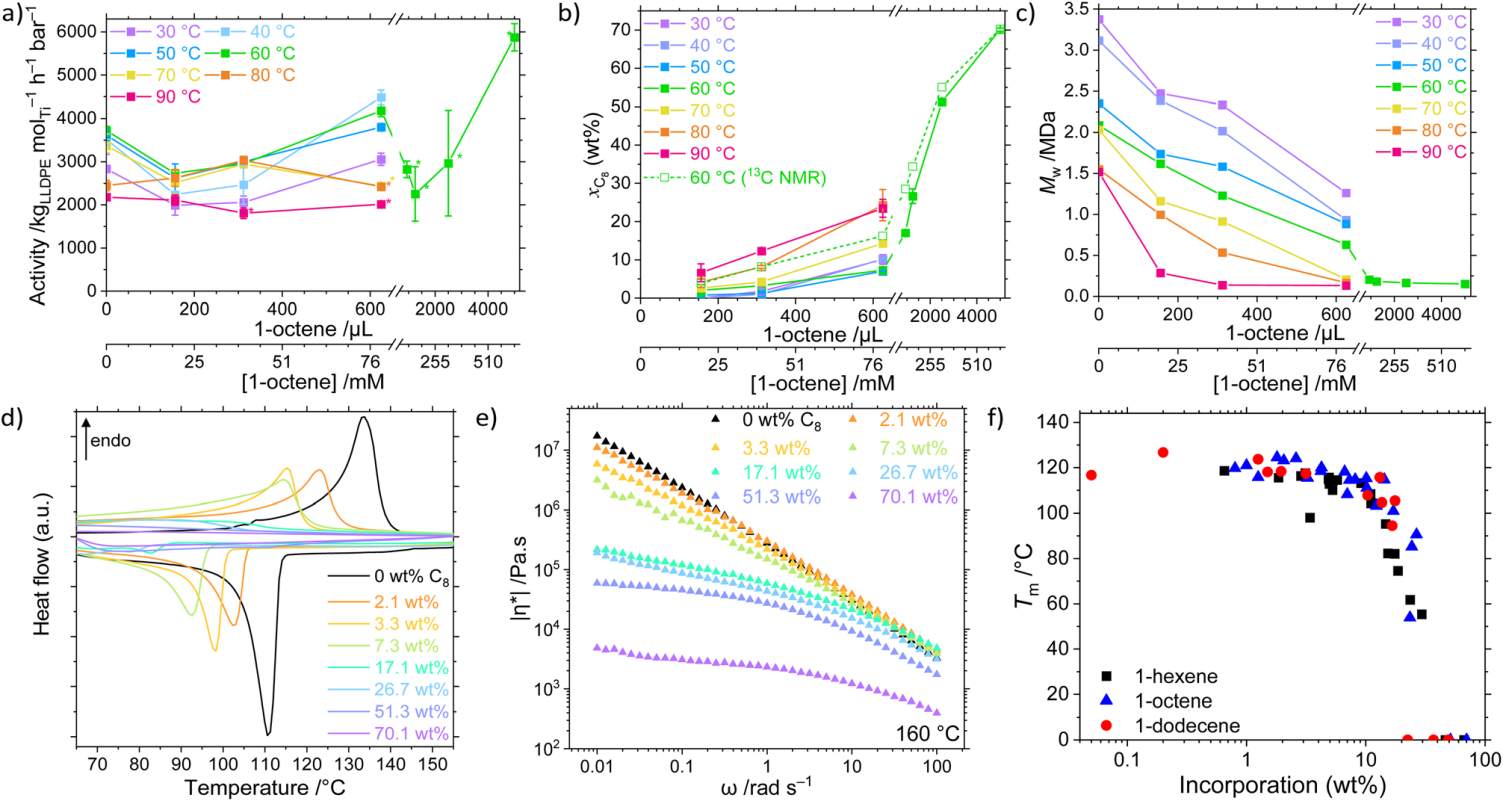
(a) Mean copolymerisation activity of 1/TIBA as a function of polymerisation temperature (30 ≤ *T*_p_ ≤ 90 °C) and 1-octene concentration (0 ≤ *V*_LAO_ ≤ 5 mL; 0 ≤ *c* ≤ 637 mM), (b) mean comonomer incorporation, and (c) weight-average molecular weight (*M*_w_) of LLDPE-C8. Comonomer incorporation determined from GPC-IR or quantitative high temperature ^13^C{^1^H} NMR spectroscopy. Error bars shown at one standard deviation. Asterisk (*) denotes gel formation. (d) Differential scanning calorimetry plots of LLDPE-C8 (20 K min^−1^, second heating and cooling shown), and (e) complex viscosity, η* (measured at 160 °C, 0.1% strain), of LLDPE-C8 synthesised at 60 °C as a function of 1-octene incorporation (GPC-IR). (f) Melting temperature of LLDPE-C*n* (*n* = 6, 8, 12) as a function of incorporation (GPC-IR). Polymerisation conditions: 10 mg 1, 2 bar ethylene, 50 mL hexanes (total volume), 0 ≤ *V*_LAO_ ≤ 5 mL, 150 mg TIBA, 30 ≤ *T*_p_ ≤ 90 °C, and 30 minutes.

The catalyst system 1/TIBA was employed in the copolymerisation of ethylene with higher α-olefins, namely 1-hexene, 1-octene, and 1-dodecene, and the effects of both polymerisation temperature and comonomer concentration were investigated (Fig. 2, Table S2 and Fig. S1–S9†). Above a threshold temperature *T*_p_, a positive comonomer effect is observed for all copolymerisations. The observed increase in activity upon addition of a comonomer relative to homopolymerisation is common, and several possible attributions have been proposed in the literature including reduced activation energies, increased number of active sites, diffusion, accelerated fragmentation, and solubility.^60–64^ In this system, the low temperature behaviour is attributed to a larger energetic barrier for LAO insertion relative to ethylene. Furthermore, at sufficiently high LAO incorporation, the LLDPE becomes soluble in hexanes – in these cases (high temperature and [LAO]), monomer diffusion becomes rate limiting, and mass-transport effects outweigh the potential increase in activity. The strongest volumetric comonomer effect was observed with 1-dodecene; at 5000 μL LAO and *T*_p_ = 60 °C, the respective activities for C6, C8, and C12 copolymerisation are 30, 58, and 113% higher compared to the homopolymerisation, up to 7930 kg_LLDPE_ mol_Ti_^−1^ h^−1^ bar^−1^ (Table 1).

**Table 1:** Selected results for the copolymerisation of ethylene and linear α-olefins using the PHENI* catalyst 1/TIBA. Data for all copolymerisation may be found in Table S2. Polymerisation conditions: 10 mg 1, 2 bar ethylene, 50 mL hexanes (total volume), 0 ≤ *V*_LAO_ ≤ 5 mL, 150 mg TIBA, and 30 minutes. Comonomer incorporation determined by GPC-IR. Complex viscosity measured at 160 °C and *ω* = 1 rad s^−1^

*n*	[LAO]/mM	*T*_p_/°C	Activity/kg_LLDPE_ mol^−1^ h^−1^ bar^−1^	*M*_w_/kDa	*Đ*	*x*_LAO_ (wt%)	*T*_m_/°C	*α* (%)	|η*|/kPa s
—	0	60	3720	2088	5.2	0	133	78	280
8	20	60	2730	1619	4.2	2.1	123	63	300
8	40	60	2950	1231	4.7	3.3	116	45	220
8	80	60	4180	632	4.2	7.3	115	40	150
8	120	60	2820	206	3.0	17.1	101	27	59
8	160	60	2250	183	3.1	26.7	91	10	43
8	320	60	2960	166	2.5	51.3	—	Amorph.	27
8	640	60	5880	152	2.5	70.1	—	Amorph.	2
8	80	30	3060	1262	4.3	10.1	115	34	*n.d.*
8	80	40	4490	933	3.9	10.2	111	34	*n.d.*
8	80	50	3800	885	4.5	7.0	108	32	*n.d.*
8	80	70	2440	209	4.2	14.3	115	46	*n.d.*
8	80	80	2420	167	3.4	24.3	85	22	*n.d.*
8	80	90	2010	133	3.2	23.5	54	18	*n.d.*
6	50	60	4220	1071	4.9	5.0	113	20	260
6	100	60	2540	182	3.0	15.3	82	22	40
6	400	60	3040	162	2.7	46.5	—	Amorph.	23
12	56	60	6000	980	5.1	3.2	117	36	240
12	113	60	3180	206	3.1	22.5	—	Amorph.	39
12	451	60	7930	177	2.5	49.3	—	Amorph.	1

Williams *et al.* have recently reported activity enhancements in E/C6 copolymerisation activity when using the permethylindenyl (η^5^-C_9_Me_6_,I*) complex ^Me_2_^SB(^^*t*^Bu^N,I*)TiCl_2_ compared to the analogous Cp*-based CGC ^Me_2_^SB(^^*t*^Bu^N,Cp*)TiCl_2_, though lower comonomer incorporations were reported for the I* complex.^56^ Copolymerisation activity of 1 at *T*_p_ = 70 °C [C6] = 50 mM (4000 kg_LLDPE_ mol_Ti_^−1^ h^−1^ bar^−1^) is comparable to the I* CGC (4400 kg_LLDPE_ mol_Ti_^−1^ h^−1^ bar^−1^) and greater than that of Cp* CGC (2260 kg_LLDPE_ mol_Ti_^−1^ h^−1^ bar^−1^) when supported on sMAO and tested under identical conditions.^65^ Albeit in less comparable conditions, the indenyl PHENICS complex ^Me_2_^SB(^^*t*^Bu,Me^ArO,Ind)TiCl_2_/TIBA/[PhNMe_2_H][BAr^F^_4_] has been reported with a moderate solution-phase activity of 8700 kg_LLDPE_ mol_Ti_^−1^ h^−1^ bar^−1^ ([C6] = 96 mM; *T*_p_ = 40 °C; 6 bar).^45^ In preliminary high-throughput screening, we found a 19-fold increase in activity with 1/TIBA compared to the indenyl complex sMAO–^Me_2_^SB(^^*t*^Bu,Me^ArO,Ind)TiCl_2_/TIBA (3004 *c.f.* 157 kg_LLDPE_ mol_Ti_^−1^ h^−1^ bar^−1^; [C6] = 400 mM, *T*_p_ = 40 °C, 8.3 bar; Table S1†).

Compared with the sterically expanded zirconocene reported by Irwin *et al.*,^47^1/TIBA does not show linearly increasing activity as a function of [LAO] but notable enhancements are seen at dramatically lower concentrations, suggestive of a uniquely “monomer-agnostic” catalyst.

### LAO enchainment efficiency

IR and quantitative high-temperature ^13^C{^1^H} NMR spectroscopy were used to determine copolymer composition (Fig. S10–S12 and S21–S26†). The values for comonomer incorporation calculated from GPC-IR are in excellent agreement with those obtained from NMR spectroscopy (*R*^2^ = 0.9072; Fig. S27†).^66^ In general, an increased temperature of polymerisation results in increased comonomer incorporation for a given concentration, as a result of being better able to overcome the larger barrier to comonomer coordination. At a C8 concentration of 40 mM, incorporation increases from 1.8 wt% at *T*_p_ = 30 °C to 12.3 wt% at 90 °C, with comparable trends observed for the C6 and C12 regimes. Analysis of the ^13^C{^1^H} NMR spectra at the triad level (Tables S3–S5†), based on the assignments of Galland *et al.*,^33^ reveals that the E/C6 copolymers measured have a tendency towards alternating microstructures. Essentially no [HHH] was detectable at any incorporation and up to 59% [EHH] was measured for LLDPE with a C6 incorporation of 52 mol%. By contrast, E/C8 copolymers appear to exhibit a more blocky microstructure, with the [OOO] triad becoming dominant at large incorporations, up to 30% at a C8 incorporation of 37 mol%. Conversely, essentially no [EOO] or [EOE] triads were detectable at any composition.

Using the Fineman–Ross method,^67^ and taking the concentration of ethylene in hexanes according to Kissin's equation, 
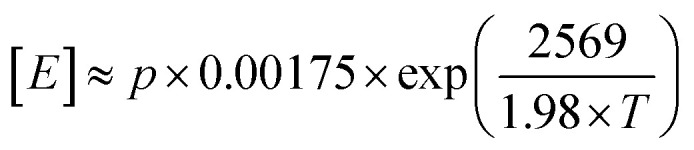
 for partial pressure, *p*, in bar and absolute temperature *T*,^68^ it is possible to estimate the reactivity ratios (Fig. S28–S30†). At *T*_p_ = 60 °C, and including only results with soluble polymers (for a consistent kinetic regime) estimates were calculated: for E/C6 copolymerisation, *r*_E_ = 10 and *r*_C6_ = 0.26 (*r*_E_ × *r*_C6_ = 2.7); for E/C8, *r*_E_ = 17 and *r*_C8_ = 0.73 (*r*_E_ × *r*_C8_ = 13); and for E/12, *r*_E_ = 13 and *r*_C12_ = 0.03 (*r*_E_ × *r*_C12_ = 0.42). This is consistent with the formation of random (*r*_E_ × *r*_C6_ ≈ 1) E/C6 copolymers, and blocky (*r*_E_ × *r*_C8_ > 1) E/C8 copolymers as observed by ^13^C NMR spectroscopy.^69^ This may have interesting implications in the material properties, since olefinic block copolymers having hard and soft segments are highly desirable thermoplastic elastomers and compatibilisers.^70–73^ Moreover, the relatively small values of *r*_E_ and large values for *r*_LAO_ (*c.f.* (SBI)ZrCl_2_*r*_E_ = 25, *r*_C6_ = 0.016;^74^ Dow-Exxon CGC *r*_E_ = 7.90, *r*_C8_ = 0.10)^75^ are indicative of PHENI* having a very high comonomer affinity. Indeed, Irwin's “sterically expanded” fluorenyl-derived CGC displayed a *r*_C10_ value of 0.49, which was claimed by the authors to be the largest for any *r*_α-olefin_ reported to date.^49^ Notwithstanding the assumptions of the Fineman–Ross method, and statistical uncertainties in the linear regression, there is strong evidence to suggest that the PHENI* catalyst 1 has a very high comonomer affinity, with *r*_LAO_ comparable to or greater than the “sterically expanded” complex, and among the highest of previously reported molecular polyolefin catalysts.

Of the available data for slurry-phase sMAO-supported catalysts tested under the same conditions as 1, both the classic Cp* CGC and its I* analogues incorporate 1-hexene far less efficiently that PHENI*. At *T*_p_ = 70 °C and [C6] = 100 mM, incorporations of 3.5 mol% (sMAO–^Me_2_^SB(^^*t*^Bu^N,Cp*)TiCl_2_), 2.4 mol% (sMAO–^Me_2_^SB(^^*t*^Bu^N,^3-Et^I*)TiCl_2_), and 2.1 mol% (sMAO–^Me_2_^SB(^^*t*^Bu^N,I*)TiCl_2_) are compared with 6.6 mol% (1) in the current study.^65^ In comparison to reported high comonomer efficiency titanium aryloxide and ketimide post-metallocenes, Nomura *et al.* reported up to 43 mol% C6 incorporation using Cp*TiCl_2_(ODipp) ([C6] = 1450 mM, 5 bar; *r*_E_ = 2.29, *r*_C6_ = 0.13), up to 40 mol% with Cp*TiCl_2_(N=C^*t*^Bu_2_) ([C6] = 2010 mM, 4 bar; *r*_E_ = 6.1, *r*_C6_ = 0.085), and up to 60 mol% using the CGC complex (^Me_2_^SB(^^*t*^Bu^N,Cp*)TiCl_2_) ([C6] = 1450 mM, 5 bar; *r*_E_ = 3.42, *r*_C6_ = 0.29).^76,77^ Dankova *et al.* reported a series of unbridged 2-arylindenyl metallocenes with high comonomer selectivity, with bis(3′,5′-di-*tert*-butyl-2-phenylindenyl)HfCl_2_ incorporating 1-hexene as well as the CGC, up to 48 mol% in neat 1-hexene ([C6] = 8000 mM, 13 bar).^78^

The virtually linear dependence of incorporation on LAO concentration is suggestive that the rate of insertion of both monomers is greater than their rates of diffusion to the active site at 2 bar pressure. Such monomer-agnostic diffusional control allows for much greater comonomer incorporations at lower initial concentrations than are commonly seen in the literature, up to 67 wt% (40 mol%) C6 at 800 mM, 70 wt% (37 mol%) C8 at 637 mM, and 49 wt% (14 mol%) C12 at 451 mM. By contrast, forcing conditions (low ethylene pressure and neat 1-hexene) are typically required to produce comparably-incorporated LLDPE using existing high efficiency catalysts such as bis-indenyl hafnocenes and CGC.^78^ In addition to this ability to incorporate very large quantities of comonomer, it also enables a wide scope for facile tuneability of intermediate incorporations.

For a given concentration, molar comonomer enchainment was found to be most efficient for 1-octene which is attributed to the slightly greater electron density compared with 1-hexene. At *T*_p_ = 60 °C incorporations of 22, 21 and 14 mol% are reported at concentrations of 400, 319, and 451 mM respectively for C6, C8, and C12. The relatively less efficient incorporation of 1-dodecene than either C6 or C8 is indicative of steric constraints becoming influential. At lower temperatures (*T*_p_ ≤ 50 °C), minimal C12 incorporations are observed, consistent with a sterically-induced increased energy barrier to comonomer insertion. However, the greater molecular weight of the C12 monomers results in a larger wt% incorporation for a given mol% which is likely to result in more significant perturbations to the physical polymer properties.

In addition to the overall comonomer incorporation, the branching density, calculated from GPC-IR as SCB/1000TC, provides an insight into the distribution and uniformity of polymer composition as a function of molecular weight. In this case, the traces are consistent with uniform copolymer composition and sample homogeneity, typical of well-controlled single-site molecular catalysts (Fig. S10–S12†).^66,79^

### Characterisation of LLDPE copolymers

In addition to the expected decrease in molecular weight with increasing temperature, a strong negative dependence of *M*_w_ on [LAO] was determined (Fig. 2c). This is because olefinic comonomers can act as chain transfer agents, higher concentrations of which increase the rate of transfer relative to propagation.^80^ It has also been shown *in silico* that the copolymer has a lower energy barrier for β-elimination (4.7 kcal mol^−1^) than the homopolymer (6.2 kcal mol^−1^), resulting in decreased molecular weights.^63^ Notably, though, this behaviour appears to be bounded in the PHENI*/E/LAO system, with high molecular weight copolymers *M*_w_ ≥ 128 kDa produced under all conditions, remining roughly invariant as a function of incorporation for values >10 wt%. This is relatively unusual, with classical CGC catalysts producing low molecular weight copolymers at high incorporations (23 kDa at 25 mol% C8) though catalysts producing high molecular weight copolymers have been developed.^35^ At lower incorporations, ultra-high molecular weight LLDPEs are obtained. The synthesis of comparatively high molecular weight LLDPE with tuneable and potentially large SCB densities is a significant feature of the PHENI* catalyst.

The physical and thermal properties of the synthesised LLDPEs depend on *M*_w_ and LAO incorporation, and therefore indirectly on the synthesis conditions. The increase in short-chain branching density afforded by LAO incorporation is expected to reduce the efficiency with which the polymer chains can pack together, reducing crystallinity and decreasing the melting point of the LLDPE relative to HDPE.^29,81^ As LAO incorporation increases, both the melting point (*T*_m_) and crystallinity (*α*) of the LLDPE decreases, eventually forming amorphous materials that exhibit elastomeric or gel-like characteristics (Fig. 2d and S13–S16†). The UHMWPE homopolymer synthesised at 60 °C had a *T*_m_ of 133 °C and crystallinity of 78%, which is reduced to 91 °C and 10% respectively at a [C8] concentration of 159 mM, corresponding to an incorporation of 26.7 wt% (by ^13^C NMR spectroscopy). At still greater incorporations, above 40 wt%, LLDPE was found to be amorphous by DSC. By rheology it was shown that the melt-phase viscosity decreases as branching content increases (Fig. 2e and S17–S19†). When measured at 160 °C and an angular frequency, *ω*, of 1 rad s^−1^, UHMWPE had a complex viscosity, |η*|, of 2.8 × 10^5^ Pa s, decreasing to 4.3 × 10^4^ Pa s at a C8 incorporation of 26.7 wt% (*α* = 10%) and 2.3 × 10^3^ Pa s at 70.1 wt% (Fig. 2e). This can also be related to an increase in tan(*δ*) as comonomer incorporation increases, with tan(*δ*) > 1 indicative of liquid-like behaviour dominated by viscous flow. This shows that beyond the amorphous limit of DSC detection, increasing SCB content continues to modify the physical properties of LLDPE by enhancing chain mobility.

Notably, for a given incorporation, there appears to be little correlation between either the thermal or rheological properties and the identity of the LAO comonomer (Fig. 2f and S20†).^26,82^ In this study, the SCB are of insufficient length for side chain crystallisation effects to become apparent.^12^ The physical properties of the copolymers are principally a function of the overall incorporation rather than any one experimental factor. This allows independent control of otherwise highly coupled parameters such as *M*_w_ and *T*_m_ which both depend on *T*_p_ and [LAO]. As a result, the PHENI*/E/LAO system offers virtually unprecedented tuneability across an extremely wide scope of LLDPEs of varying chemical, thermal, and physical properties. Though beyond the scope of this study, the reduction in crystallinity and melting point is expected to have a profound effect on many other physical and mechanical properties of the polymers.^83,84^ The ability to access a wide range of incorporations and physical characteristics from a single catalyst system is of significant industrial relevance.

### Multivariate regression modelling of E/LAO copolymerisation

The unique monomer-agnostic behaviour of the PHENI* catalyst allows a high degree of tunability and control over polymer composition. This presents an opportunity to synthesise “designer” polyolefins, where a pre-defined set of material properties can be targeted by the simple adjustment of comonomer feed and temperature.

Modelling of ethylene polymerisation systems generally focuses on quantitative structure–activity relationships (QSAR) informed by density functional theory (DFT) calculations on the precatalysts.^85–87^ This can give useful chemical and mechanistic insights, and inform future catalyst development.^88^ Beyond fundamental chemical research, the ultimate goal of such methodology is to construct a model that is able to predict experimental parameters to tune polymerisation properties. In real-world systems, many factors beyond precatalyst QSAR are significant, such as morphology, shear forces, diffusion, mixing, impurities, scavengers, and cocatalysts. Furthermore, the structures of components such as MAO and sMAO are not fully elucidated,^89,90^ and even the oxidation state of the active species is debated.^91^ Recently, models using multiple linear regression have been proposed in combination with large datasets obtained by high-throughput experimentation with reasonable explanatory and predictive abilities.^92^

Multivariate data analysis was carried out on the PHENI*/E/LAO copolymerisation dataset, with the aim of establishing a predictive model for the synthesis of designer polymers. To our knowledge this is the first application of such a regression model to olefin copolymerisations in the peer-reviewed literature.

**Fig. 3:**
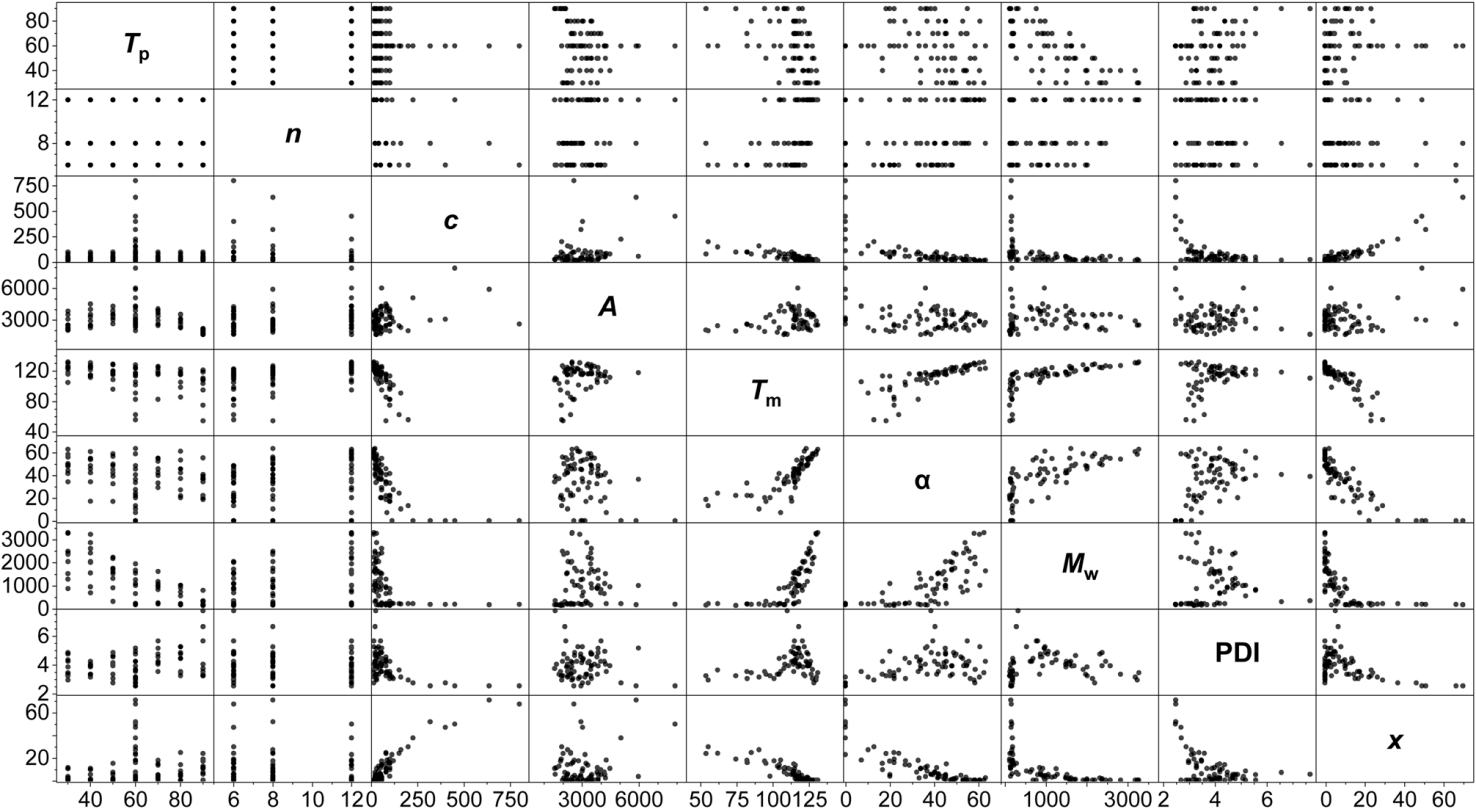
Scatterplot matrix of the PHENI*/E/LAO dataset, showing 2-dimensional scatter plots of pairs of variables. Temperature of polymerisation (*T*_p_; °C); LAO length (carbon number, *n*); [LAO] (*c*; mM); activity (*A*; kg_LLDPE_ mol^−1^ h^−1^ bar^−1^); melting point (*T*_m_; °C); crystallinity (*α*; %); molecular weight (*M*_w_; kDa); dispersity (PDI, *Đ* = *M*_w_/*M*_n_); LAO incorporation (*x*; wt%). *N* = 75.

A standard full-factorial model (M1) was used, regressing the response variables (activity *A*, polymer melting point *T*_m_, crystallinity *α*, molecular weight *M*_w_, dispersity PDI, *Đ* and comonomer incorporation *x*) against full-factorial polynomial combinations of the explanatory variables up to quadratics: temperature of polymerisation *T*_p_, LAO length *n*, and LAO concentration *c* (*T*_p_, *c*, *n*, *T*_p_^2^, *c*^2^, *n*^2^, *T*_p_*n*, *T*_p_*c*, *cn*; Fig. 3). On the basis of likelihood ratio tests, *T*_p_ and *c* explain much of the variation, with −log_10_(*p*-values) of 33.0 and 24.9 respectively, and all of the polynomial terms apart from *n*^2^ have significant relationships at the 0.01 level of hypothesis testing. The model is predictive with *R*^2^ values of: *A* (0.65), *T*_m_ (0.85), *α* (0.81), *M*_w_ (0.96), PDI (0.50), and *x* (0.85).

Modelling of this kind enables the delineation of interrelated variables. Incorporation is determined almost linearly by *c*, alongside a contribution from *T*_p_. While *T*_m_ is determined principally by the temperature-concentration couple, crystallinity depends more strongly on the side chain length, with the predictors *nc*, *n*^2^ and *T*_p_*n* all having statistically significant contributions. This is consistent with physical expectations: increased comonomer concentration (and therefore, incorporation) increases the degree of branching, which reduces the intermolecular forces between polymer chains and lowers the melting point.^93^ The branches are generally excluded from the crystalline lamellae, disrupt chain folding and lead to defective crystallisation,^94^ with the length of the side chain influencing crystallinity.^95^

The anticipated dependency of *M*_w_ on both *T*_p_ and *c* is reflected in the model, and of the cross terms, *T*_p_*n* has the greatest effect, showing that temperature-chain length coupling is a more important factor than concentration-chain length. This is suggestive of a mechanistic interpretation, with the larger energy barriers associated with larger monomers interacting with the thermal energy in the system.

### Towards designer polyolefins

That the relatively simple regression model (M1) captures so much of the chemical and physical behaviour of this highly non-trivial reaction system demonstrates not only the power of relatively large datasets for the delineation of interrelated variables, but also the potential ability to leverage the tuneability of the PHENI* catalyst system towards parameter-space optimisation.

**Fig. 4:**
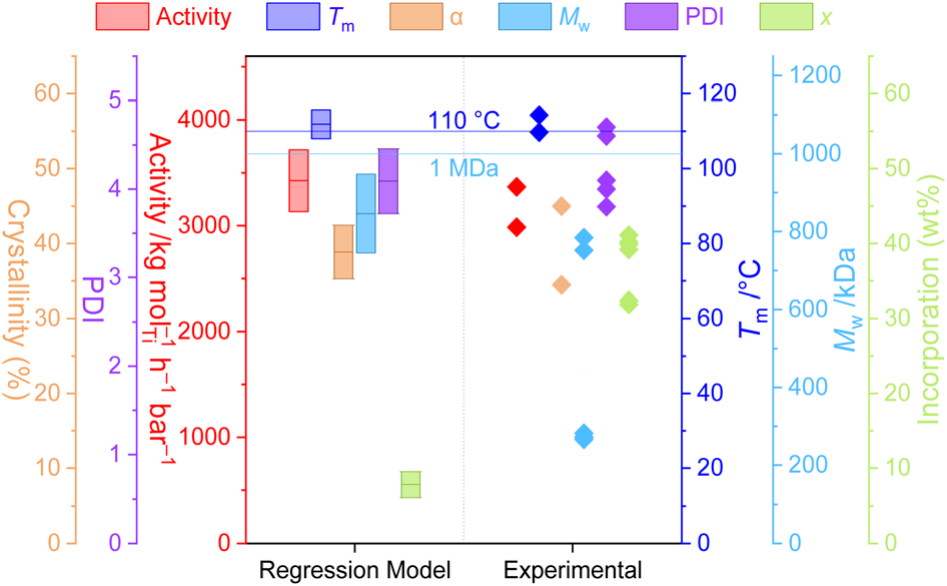
Polymerisation parameters of P1 predicted by the regression model M1 (95% confidence intervals shown) and experimental results. Horizontal lines indicated the target values which determined the optimisation functions for calculating experimental conditions. Polymerisation conditions: 10 mg 1, 50 mL hexanes, 150 mg TIBA, 2 bar ethylene, 30 minutes, 61 °C, [C8] = 55.5 mM.

To explore this, the model was optimised with respect to three sets of “designer” copolymer properties, with the model converging on reaction conditions expected to produce the desired materials. A detailed discussion of methodology and results may be found in the ESI.† There was variable agreement between the desired, predicted, and experimental values, though the polymer properties that were interpolated within the model data were well predicted. In particular, sample P1 (optimised towards the desired properties of *T*_m_ = 110 °C and *M*_w_ = 1 MDa) resulted in very well predicted values for activity (M1 3424 kg mol^−1^ h^−1^ bar^−1^; expt. 3180 ± 270 kg mol^−1^ h^−1^ bar^−1^), melting point (M1 112 °C; expt. 112 ± 3 °C), crystallinity (M1 39%; expt. 40 ± 7%), molecular weight (M1 846 kDa, expt. 606 ± 281 kDa), and dispersity (M1 4.1; expt. 4.1 ± 0.4) (Fig. 4). Additionally, the polymer melting temperature closely fitted the value that was desired through programmed synthesis. The relatively large uncertainty in measured *M*_w_ results for the reaction being at the boundary of the soluble and insoluble regimes, with poor reproducibility between runs. Surprisingly, and despite the well-predicted macroscopic thermal properties, the incorporation itself was relatively poorly predicted in this case, despite being one of the simplest, best correlated aspects of the model. The generally good agreement between the desired, predicted, and experimental parameters highlights the power and utility of statistical modelling for the synthesis of designer polymers.

Sample P2 was optimised with respect to four parameters simultaneously which led to poorer convergence of the model, though the experimentally measured parameters, including incorporation, were generally well predicted by M1 (Fig. S33†). Finally, sample P3 was extrapolated beyond the scope of the dataset, with a poor optimisation and generally inaccurate predictions highlighting the limitations of this method much beyond the property-space of the data used to construct M1.

This pilot study demonstrates the underlying utility of multivariate modelling, combined with relatively large datasets, in delineating and predicting the outcomes of complex chemical reactions. It is envisaged that including mechanical and material characterisation in the modelling would dramatically expand the scope of this methodology towards the application-directed precision synthesis of “designer” polyolefins. The utility of such a methodology may have far-reaching applications in many areas of chemistry.

## Conclusions

The PHENI* catalyst has demonstrated excellent catalytic performance across a range of olefinic copolymerisations, having one of the highest LAO affinities reported. The PHENI*/E/LAO system offers virtually unprecedented tuneability across an extremely wide scope of LLDPEs of varying chemical, thermal, and physical properties. In all cases, composition is determined by the concentration of monomers, highlighting the desirable and unusually monomer-agnostic behaviour of this catalyst.

The potential of synthesising industrially relevant designer polyolefins using a single catalyst based on statistical models has been demonstrated in principle. The advent of parallelised high-throughput experiments now enables efficient dataset acquisition, and the inclusion of additional parameters such as pressure, and mechanical and material properties would further enable a dramatically expanded scope of tunability and control, with the ultimate goal of entirely application-directed “programmable and predictable” synthesis.^35^

## Data availability

Data for this paper, including polymerisation data, polymer characterisation data, and regression modelling, are available in the ESI.†

## Author contributions

C. G. C. R.: investigation, conceptualisation, formal analysis, writing – original draft, reviewing & editing. L. J. M.: supervision, writing – reviewing & editing. J.-C. B.: conceptualisation. Z. R. T.: conceptualisation, project administration, supervision, writing – reviewing & editing. D. O. H: funding acquisition, project administration, supervision, writing – reviewing & editing.

## Conflicts of interest

There are no conflicts to declare.

## Supplementary Material

SC-015-D3SC04861F-s001
