# Supplementary material for: Towards designer polyolefins: highly tuneable olefin copolymerisation using a single permethylindenyl post-metallocene catalyst
Source: Chem Sci. 2023 Dec 6;15(1):250–8. doi: 10.1039/d3sc04861f (PMC10731910; doi:10.1039/d3sc04861f)
Supplement: SC-015-D3SC04861F-s001 [file SC-015-D3SC04861F-s001.pdf]

Electronic Supplementary Material (ESI) for Chemical Science.
This journal is © The Royal Society of Chemistry 2023

Supplementary Material

Towards designer polyolefins: highly tuneable olefin copolymerisation using a single permethylindenyl post-metallocene catalyst

Clement G. Collins Rice, Louis J. Morris, Jean-Charles Buffet, Zoë R. Turner, and Dermot O'Hare
Chemistry Research Laboratory, University of Oxford, 12 Mansfield Road, Oxford, OX1 3TA, U.K.

Table of contents

1. General details and instrumentation S2
2. Experimental details S3
3. High throughput screening S5
4. Copolymerisation data tables S6
5. Copolymerisation graphical results S10
6. Gel-permeation chromatography S19
7. Differential scanning calorimetry S22
8. Rheology S28
9. NMR spectroscopy S29
10. Fineman-Ross analysis S34
11. Multiple linear regression S37
12. Designer LLDPE S42
13. References S47

1. General details and instrumentation

General procedures. Air- and moisture-sensitive compounds were manipulated under an inert atmosphere of nitrogen, using standard Schlenk line techniques[1] on a dual manifold vacuum/nitrogen line or in an MBraun Labmaster 100 glovebox.

Pentane, hexanes, toluene and benzene were dried using an MBraun SPS 800 solvent purification system, stored over a potassium mirror, and degassed under partial vacuum before use. Anhydrous dichloromethane was dried using an MBraun SPS 800 system, stored over pre-activated 3 Å molecular sieves and degassed under partial vacuum before use. Tetrahydrofuran was distilled from sodium/benzophenone, stored over pre-activated 3 Å molecular sieves and degassed under partial vacuum before use.

Deuterated solvents were dried over potassium metal (benzene-d_6, and toluene-d_8) or CaH_2 (chloroform-d, pyridine-d_5, and tetrahydrofuran-d_8) and refluxed under reduced pressure for 5 days, distilled under static vacuum, freeze-pump-thaw degassed three times and stored over pre-activated 3 or 4 Å molecular sieves. Chloroform-d and 1,1,2,2-tetrachloroethane-d_2 were used as supplied for samples which were not air- and moisture-sensitive.

Solution NMR spectroscopy. NMR spectra were recorded on either a Bruker Avance III HD NanoBay NMR (9.4 T, 400.2 MHz), a Bruker Avance III NMR (11.75 T, 499.9 MHz), a Bruker NEO 600 (14.1 T, 600.4 MHz) with a broadband helium cryoprobe, or a Bruker Avance NMR (11.75 T, 500.3 MHz) with a ^{13}C-detect cryoprobe. Spectra were recorded at 298 K unless otherwise stated and referenced internally to the residual *protio* solvent resonance. Chemical shifts, δ, are reported in parts per million (ppm) relative to tetramethylsilane (δ = 0 ppm). Air-sensitive samples were prepared in a glovebox under an inert atmosphere of nitrogen, using dried deuterated solvents and sealed in 5 mm Young's tap NMR tubes. Quantitative ^{13}C NMR spectroscopy was performed using an inverse-gated ^{1}H decoupling pulse sequence, a relaxation delay of 60 s, and 2.8 mg mL^{-1} $Cr(acac)_3$ as a T_1 relaxation agent.

Gel permeation chromatography. Gel permeation chromatography (GPC) was performed by Ms Liv Thobru, Ms Sara Rund Herum, and Ms Rita Jenssen (Norner AS, Norway) on a high temperature gel permeation chromatograph with an IR5 infrared detector (GPC-IR5). Samples were prepared by dissolution in 1,2,4-trichlorobenzene (TCB) containing 300 ppm of 3,5-di-*tert*-buty-4-hydroxytoluene (BHT) at 160 °C for 90 minutes and then filtered with a 10 µm SS filter before being passed through the GPC column. The samples were run under a flow rate of 0.5 mL min^{-1} using TCB containing 300 ppm of BHT as mobile phase with 1 mg mL^{-1} BHT added as a flow rate marker. The GPC column and detector temperature were set at 145 and 160 °C respectively.

Differential scanning calorimetry. Differential scanning calorimetry was performed on a Perkin Elmer DSC 4000 System within a temperature range of 10–250 °C at a rate of 20 K min^{-1}. Polymer samples were sealed in 100 µL aluminium crucibles. An empty crucible was used as a reference, and the DSC was calibrated using indium and zinc.

Rheology. Rheology was performed on a TA Instruments Discovery HR-2 hybrid rheometer using a temperature controlled stainless steel Peltier plate and a flat parallel plate geometry (20 mm diameter) with a working gap of 1000 µm. Approximately 200 mg of material was first vacuum compression moulded into a disc (10 mm diameter) at 180 °C. Measurements were performed at 160 °C under a flow of dry nitrogen in continuous oscillation (direct strain) mode at a strain of 0.1% (within the LVE region) and a logarithmic frequency sweep was performed from 0.01–100 rad s^{-1}.

Literature preparations and commercially supplied materials. 2,3,4,5,6,7-hexamethylindene (SCG Chemicals PLC); ^{n}BuLi (1.6 M in hexanes), 4-methyl-2-*tert*-butylphenol, bromine, titanium tetrachloride, tri*iso*butylaluminium (Sigma Aldrich); and 6-bromo-2,4-di-*tert*-butylphenol (Alfa Aesar) were all used as received. 2,4-bis(α,α-dimethylbenyl)phenol (Sigma Aldrich) was recrystallised from hot ethanol before use. $Ind^{\#}Li$,[2,3] and $TiCl_4·2THF$[4] were prepared according to literature procedures. Et_3N was dried over KOH, distilled under static vacuum and freeze-pump-thaw degassed before use. Me_2SiCl_2 (Sigma Aldrich) was dried over pre-activated 3 Å molecular sieves before use. Allyl bromide (Sigma Aldrich) was washed with $NaHCO_3$ followed by distilled water and dried over $MgSO_4$. 1-Hexene, 1-dodecene (Sigma Aldrich), and 1-octene (Alfa Aesar) were dried over CaH_2, distilled under static vacuum, freeze-pump-thaw degassed three times and stored over pre-activated 3 or 4 Å molecular sieves.

PHENI* catalysts **1**–**3** were synthesised according to a literature procedure,[5] and the indenyl-PHENICS complex **4** according to a modified literature procedure.[6] Ethylene was supplied by BOC Ltd. and was passed through pre-activated molecular sieves before use. Solid polymethylaluminoxane (sMAO, 3rd generation) was supplied by SGC Chemicals PLC as a slurry in toluene which was dried under vacuum before use.

2. Experimental details

High-throughput screening was performed at Xplore s.r.l. (University of Naples Federico II) by V. Busico, R. Cipullo, L. Rongo, and A. Mingione. Polymerisation experiments were conducted in a FreeSlate Parallel Pressure Reactor (PPR) platform consisting of 48 reaction cells contained within a MBraun LabMaster glovebox, which has been described extensively elsewhere.[7] Heptane solvent (5 mL) and TIBA scavenger (10 µmol) were added to the PPRs *via* robotic syringes which were then heated to the required temperature and pressurised to 120 psi (8.3 bar) with ethylene. Pre-catalyst (0.05–0.80 mg in heptane slurry) and 1-hexene were injected with robotic syringes. The reactions were run for 1 hour or until a certain ethylene uptake was reached, after which the reactions were quenched with an excess of dry air. The polymer samples were transferred to a Genevac EZ-Plus centrifugal evaporator to remove the volatiles and then dried under vacuum overnight.

Copolymerisation of ethylene with linear α-olefins. In a typical procedure, 150 mg tri*iso*butylaluminium (TIBA) was added into a vial and 10 mL of hexanes was added. This mixture was introduced into a 150 mL Rotaflo® ampoule containing a stirrer bar and swirled around the glassware. 10 mg supported catalyst was added to the ampoule and washed in with a further 35 mL hexanes. To a sidearm the desired amount of comonomer was added and washed in with sufficient hexanes to keep the total reaction volume at 50 mL. The ampoule was sealed, cycled onto a Schlenk line, and degassed under reduced pressure. It was cycled a further two times using ethylene as a purge gas while the vessel was brought to temperature in a thermostatic oil bath with the stirring set at 1000 rpm. The stopcock was opened to ethylene at a pressure of 2 bar simultaneously with the introduction of the comonomer solution from the sidearm and the timer was started. On completion of the run, the vessel was degassed under partial vacuum then either filtered on a sintered glass frit (porosity 3), and washed with 2 x 25 mL pentane, or precipitated by decanting into a 500 mL round-bottomed flask containing dilute aqueous hydrochloric acid and rotary evaporation of the organic solvent, from which the polymer could be manually separated. Polymers were dried under vacuum until constant weight. All runs were carried out at least in duplicate to ensure reproducibility.

Ethylene/1-hexene copolymer.

$^{13}C\{^1H\}$ NMR (151 MHz, 1,1,2,2-tetrachloroethane-d_2, 403 K): δ 38.20, 34.60, 34.21, 30.45, 29.95, 29.56, 27.29, 23.37, and 14.18 ppm.

Ethylene/1-octene copolymer.

$^{13}C\{^1H\}$ NMR (151 MHz, 1,1,2,2-tetrachloroethane-d_2, 403 K): δ 38.21, 34.59, 32.20, 30.45, 29.95, 27.28, 22.87, and 14.15. ppm.

Ethylene/1-dodecene copolymer.

$^{13}C\{^1H\}$ NMR (151 MHz, 1,1,2,2-tetrachloroethane-d_2, 403 K): δ 38.23, 34.60, 32.19, 30.45, 29.96, 29.56, 27.29, 22.86, and 14.14 ppm.

Multivariate regression analysis. Data analysis and prediction was performed using the JMP® Pro software suite.[8] A standard full factorial least squares model was used to construct models of A, T_m, α, M_w, PDI, x against T_p, c, n, T_p^2, c^2, n^2, T_pn, T_pc, and cn. Analysis of variance (ANOVA) tests the assumptions of homoscedasticity and normality of residuals. Effect test analysis shows which predictors are significant.

The prediction profiler was used with defined desirability functions to determine the experimental conditions for a defined set of copolymer properties.

3. High throughput screening

Table S1 Polymerisation data using **1**, or related supported catalysts[5] sMAO-^{Me2}SB(^{tBu,Me}ArO,I*)$TiCl_2$ (**2**), sMAO-^{Me2}SB($^{(CMe2Ph)2}$ArO,I*)$TiCl_2$ (**3**), or sMAO-^{Me2}SB(^{tBu,Me}ArO,Ind)$TiCl_2$ (**4**), performed at Xplore s.r.l.. Polymerisation conditions: 0.05–0.80 mg solid catalyst, heptane diluent (5.0 mL total volume), ethylene (8.3 bar), TIBA (10 µmol), and either 60 minutes or until 120 psi ethylene uptake. M_w and dispersity determined by GPC, 1-hexene incorporation determined by high-temperature ^{13}C NMR spectroscopy, $T_{el,max}$ determined by Crystallisation Elution Fractionation (CEF). *n.d.* indicates no data available.

Catalyst	C6 /µL	[C6] /mM	T_p /°C	Activity /kg_{LLDPE} mol^{-1} h^{-1} bar^{-1}	M_w /kDa	PDI	x_{C6} (mol%)	$T_{el,max}$ /°C
**4**/TIBA	75	120	40	60 ± 3	*n.d.*	*n.d.*	2.4	75.2/111.5
	125	200	40	110 ± 1	482	22.2	5.2	76.0/110.7
	250	400	40	160 ± 20	*n.d.*	*n.d.*	*n.d.*	*n.d.*
	125	200	60	40 ± 8	*n.d.*	*n.d.*	4.9	78.5/108.5
	250	400	60	90 ± 3	*n.d.*	*n.d.*	*n.d.*	*n.d.*
**1**/TIBA	75	120	40	1400 ± 100	*n.d.*	*n.d.*	0.4	*n.d.*
	125	200	40	1400 ± 50	2212	2.8	0.8	*n.d.*
	250	400	40	3000 ± 230	*n.d.*	*n.d.*	*n.d.*	*n.d.*
	75	120	60	3400 ± 6	*n.d.*	*n.d.*	1.5	*n.d.*
	125	200	60	1300 ± 360	*n.d.*	*n.d.*	4.5	75.7/95.9
	250	400	60	1600 ± 560	*n.d.*	*n.d.*	*n.d.*	*n.d.*
	75	120	80	480 ± 6	667	2.7	3.1	*n.d.*
**2**/TIBA	75	120	40	450 ± 1	*n.d.*	*n.d.*	0.3	*n.d.*
	125	200	40	510 ± 30	2202	2.9	0.85	*n.d.*
	250	400	40	700 ± 80	*n.d.*	*n.d.*	*n.d.*	*n.d.*
	125	200	60	650 ± 250	*n.d.*	*n.d.*	4.5	*n.d.*
	250	400	60	780 ± 10	*n.d.*	*n.d.*	*n.d.*	*n.d.*
**3**/TIBA	75	120	40	970 ± 90	*n.d.*	*n.d.*	0.5	*n.d.*
	125	200	40	1200 ± 30	1707	4.9	1.1	*n.d.*
	250	400	40	3200 ± 200	*n.d.*	*n.d.*	*n.d.*	*n.d.*

75	120	60	2400 ± 40	*n.d.*	*n.d.*	1.6	*n.d.*
125	200	60	1892 ± 100	*n.d.*	*n.d.*	4.1	84.4
250	400	60	3800 ± 1400	*n.d.*	*n.d.*	*n.d.*	*n.d.*
75	120	80	640 ± 120	945	3.0	2.9	*n.d.*

4. Copolymerisation data tables

Table S2 Polymerisation data using **1**. Polymerisation conditions: 10 mg catalyst, 2 bar ethylene, 150 mg TIBA, 30 minutes, 50 mL hexanes.

n	**LAO /µL**	**[LAO] /mM**	**T_p /°C**	**Activity /kg_{LLDPE} mol^{-1} h^{-1} bar^{-1}**	**M_w /kDa**	**PDI**	**x_{LAO}, GPC-IR (wt%)**	**x_{LAO}, ^{13}C NMR (wt%)**	**T_m /°C**	**α (%)**
6	156	25	30	2300 ± 690	2021	3.2	0.0		122.3	47
6	312	50	30	2200 ± 870	1501	3.8	3.1		117.7	44
6	625	100	30	3800 ± 1800	857	3.3	11.1		104.5	41
6	156	25	40	2400 ± 230	1987	3.3	0.0		121.5	48
6	312	50	40	2200 ± 60	1527	4.0	4.9		114.5	42
6	625	100	40	4000 ± 900	670	4.2	9.1		113.2	17
6	156	25	50	3500 ± 240	1699	3.9	0.7		118.7	48
6	312	50	50	3900 ± 20	1291	4.8	5.3		114.4	39
6	625	100	50	3200 ± 230	293	3.3	14.7		95.3	17
6	156	25	60	3400 ± 20	1516	4.2	1.9	10.3	115.6	33
6	312	50	60	4200 ± 220	1071	4.9	5.0	15.0	112.9	20
6	625	100	60	2500 ± 50	182	3.0	15.3	35.9	82.3	22
6	937	150	60	2400 ± 270	192	3.6	23.5	43.8	61.8	24
6	1250	200	60	1900 ± 650	195	2.9	29.4	53.7	55.4	13
6	2500	400	60	3000 ± 60	162	2.7	46.5	63.8	-	Amorphous
6	5000	800	60	2600 ± 700	169	2.5	66.6	76.4	-	Amorphous

6	156	25	70	3500 ± 140	1058	4.7	2.9		116.4	42
6	312	50	70	4000 ± 320	807	5.6	4.9		115.1	39
6	625	100	70	2600 ± 10	182	3.5	17.4		82.2	33
6	156	25	80	3533 ± 440	876	4.7	4.9		115.8	45
6	312	50	80	2300 ± 190	186	3.5	5.8		114.6	42
6	625	100	80	3500 ± 130	146	3.2	3.4		98.1	20
6	156	25	90	1600 ± 590	333	7.7	5.3		110.2	39
6	312	50	90	1700 ± 280	154	4.0	11.0		108.5	36
6	625	100	90	1900 ± 160	126	3.2	18.6		74.6	22

8	156	20	30	2000 ± 230	2474	4.2	0.0		126.3	56
8	312	40	30	2100 ± 150	2335	4.7	1.8		124.6	50
8	625	80	30	3100 ± 140	1262	4.3	10.1		115.3	34
8	156	20	40	2200 ± 60	2390	3.9	0.0		124.4	54
8	312	40	40	2500 ± 450	2019	3.8	1.0		121.0	45
8	625	80	40	4500 ± 170	933	3.9	10.2		111.1	34
8	156	20	50	2600 ± 330	1739	4.6	0.8		119.7	49
8	312	40	50	3000 ± 20	1583	4.1	1.3		115.8	45
8	625	80	50	3800 ± 90	885	4.5	7.0		108.2	32
8	156	20	60	2700 ± 90	1619	4.2	2.1	3.9	123.2	63
8	312	40	60	3000 ± 30	1231	4.7	3.3	8.2	115.5	45
8	625	80	60	4200 ± 140	632	4.2	7.3	16.2	114.5	40
8	937	120	60	2800 ± 190	206	3.0	17.1	28.5	101.0	27
8	1250	160	60	2300 ± 630	183	3.1	26.7	34.4	90.6	10
8	2500	320	60	3000 ± 1200	166	2.5	51.3	55.1	-	Amorphous
8	5000	640	60	5900 ± 320	152	2.5	70.1	70.3	-	Amorphous

8	156	20	70	2500 ± 260	1163	4.1	2.6		124.1	54
8	312	40	70	2900 ± 890	916	4.5	4.3		120.1	52
8	625	80	70	2400 ± 40	209	4.2	14.3		114.7	46
8	156	20	80	2600 ± 200	998	4.8	4.2		118.5	45
8	312	40	80	3000 ± 90	537	5.2	8.2		114.7	37
8	625	80	80	2400 ± 2	167	3.4	24.3		85.2	22
8	156	20	90	2100 ± 120	289	6.6	6.7		118.2	40
8	312	40	90	1800 ± 120	139	3.3	12.3		103.3	37
8	625	80	90	2000 ± 70	133	3.2	23.5		53.9	18

12	156	14	30	2500 ± 70	3285	3.4	0.0		131.3	63
12	312	28	30	2500 ± 190	3249	2.9	0.0		130.0	58
12	625	56	30	3500 ± 630	2389	4.8	1.3		123.7	49
12	156	14	40	2900 ± 70	3200	3.1	0.0		130.3	61
12	312	28	40	3300 ± 450	2839	3.3	0.2		126.7	54
12	625	56	40	3500 ± 40	2587	3.9	0.0		125.3	55
12	156	14	50	3300 ± 240	2206	2.7	0.0		128.7	59
12	312	28	50	3500 ± 160	2153	3.0	0.0		127.6	56
12	625	56	50	4300 ± 50	1499	4.0	2.0		118.4	42
12	156	14	60	3500 ± 80	1925	3.6	0.0	3.0	127.7	59
12	312	28	60	3900 ± 320	1647	3.9	0.0	2.2	124.1	48
12	625	56	60	6000 ± 280	980	5.1	3.2	7.9	117.4	36
12	937	84	60	4300 ± 870	198	3.5	17.6	24.0	105.5	7
12	1250	110	60	3200 ± 30	206	3.1	22.5	27.8	-	Amorphous
12	2500	230	60	5100 ± 240	192	2.7	37.1	42.6	-	Amorphous
12	5000	450	60	7900 ± 370	177	2.5	49.3	76.5	-	Amorphous

12	156	14	70	3700 ± 280	1598	4.0	0.0	125.7	55
12	312	28	70	3900 ± 230	970	5.1	1.5	118.1	45
12	625	56	70	3300 ± 310	230	4.1	13.1	115.7	29
12	156	14	80	2800 ± 160	990	5.2	0.0	122.0	61
12	312	28	80	3100 ± 100	697	4.4	0.1	116.7	53
12	625	56	80	2200 ± 320	200	4.4	13.7	104.7	27
12	156	14	90	2200 ± 100	777	5.6	0.0	120.7	55
12	312	28	90	1600 ± 290	148	3.7	10.4	107.8	34
12	625	56	90	1900 ± 100	128	3.5	16.7	94.5	20

5. Copolymerisation graphical results

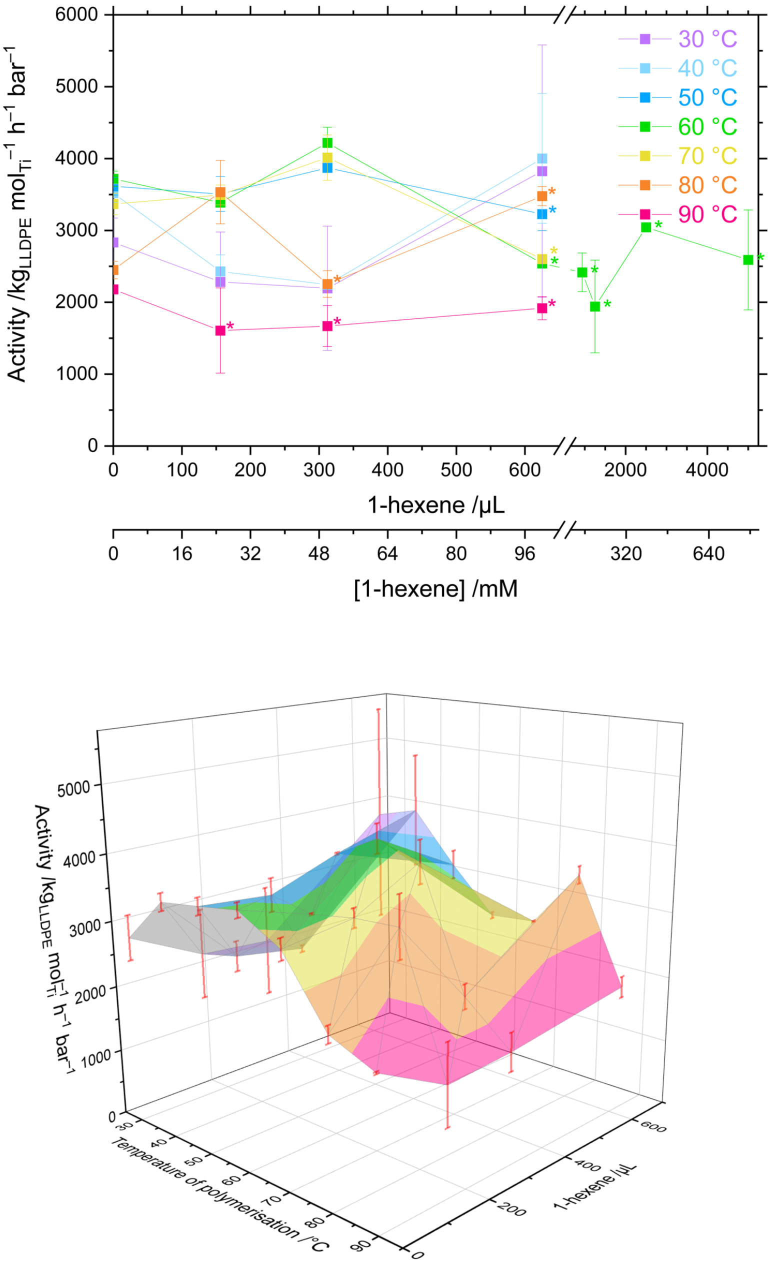

Figure S1 Mean copolymerisation activity of **1** as a function of temperature of polymerisation (30 ≤ T_p ≤ 90 °C) and 1-hexene concentration (0 ≤ V ≤ 5000 μL; 0 ≤ c ≤ 800 mM). Polymerisation conditions: 10 mg catalyst, 2 bar ethylene, 50 mL hexanes (total volume), 150 mg TIBA, and 30 minutes. Error bars shown at one standard deviation. Asterisk (*) denotes gel formation.

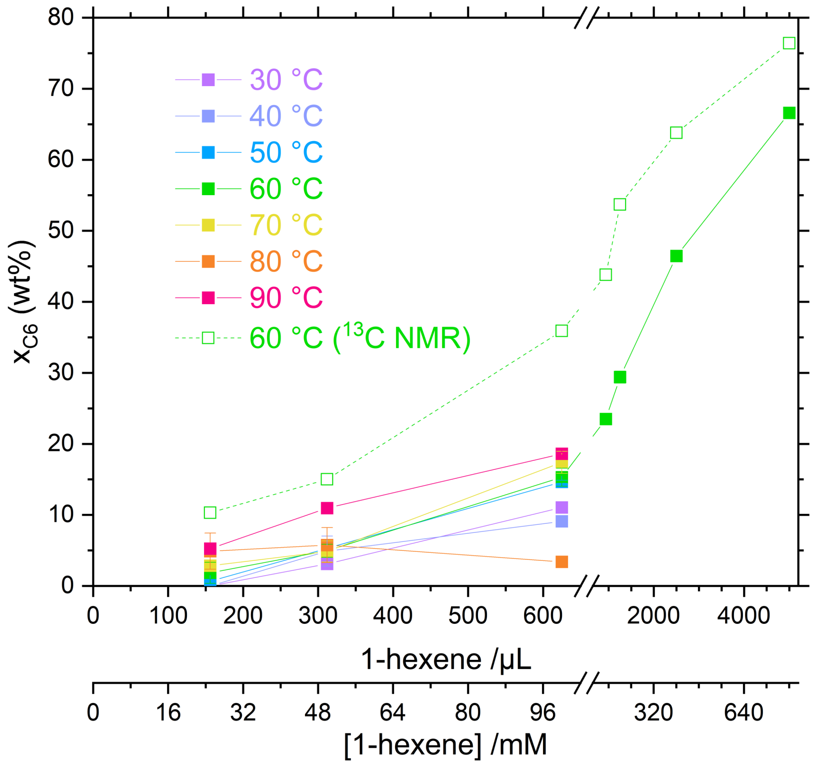

Figure S2 Mean comonomer incorporation, as a function of concentration of 1-hexene, of LLDPE-C6 synthesised by **1**/TIBA, obtained from GPC-IR or ^{13}C NMR spectroscopic measurements. Error bars shown at one standard deviation.

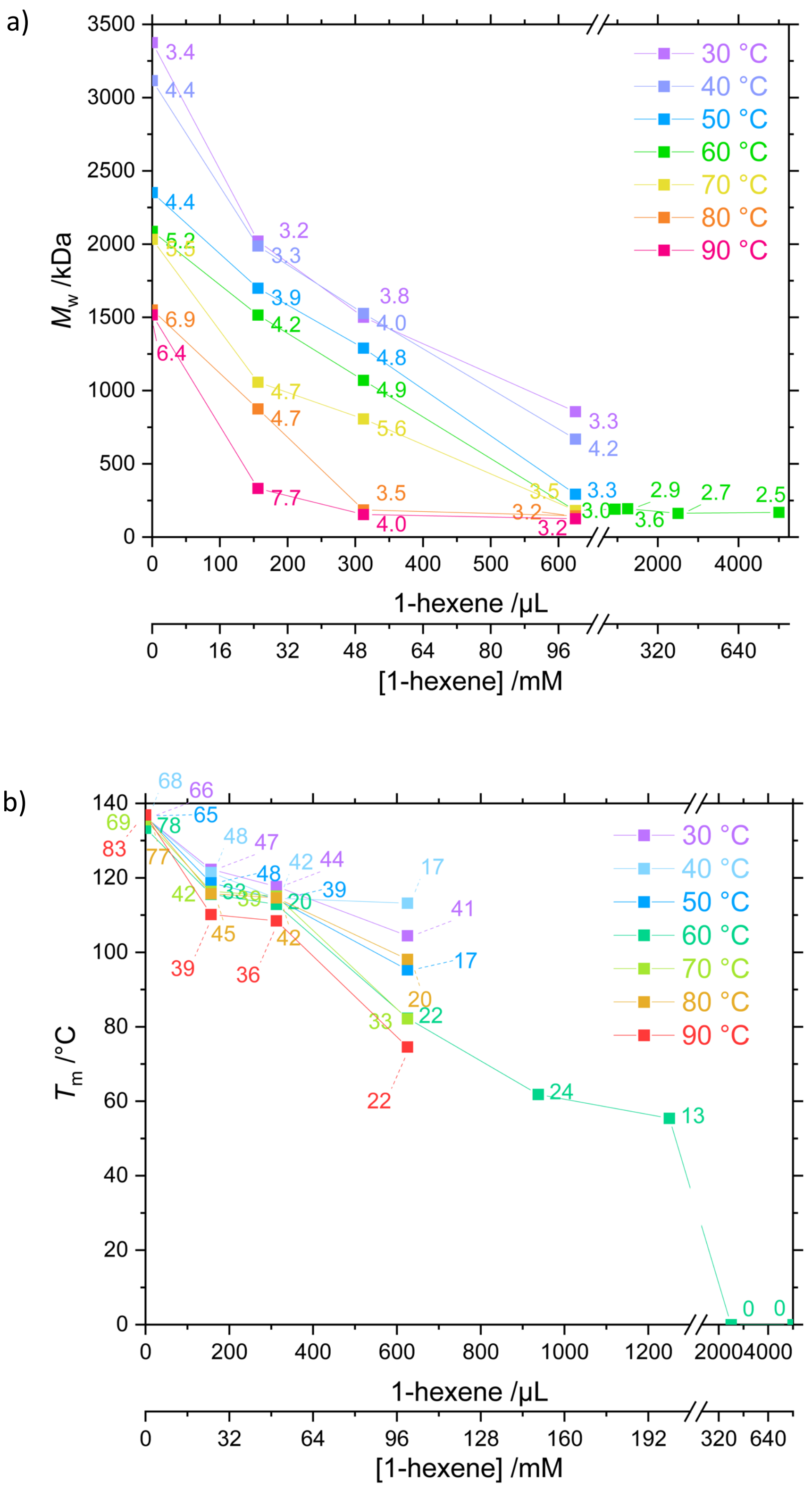

Figure S3 a) Weight-average molecular weight (with PDIs annotated) of LLDPE-C6 synthesised by **1**/TIBA as a function of temperature of polymerisation and 1-hexene concentration. b) Melting temperature (with crystallinity annotated) of LLDPE-C6 synthesised by **1**/TIBA as a function of temperature of polymerisation and comonomer concentration.

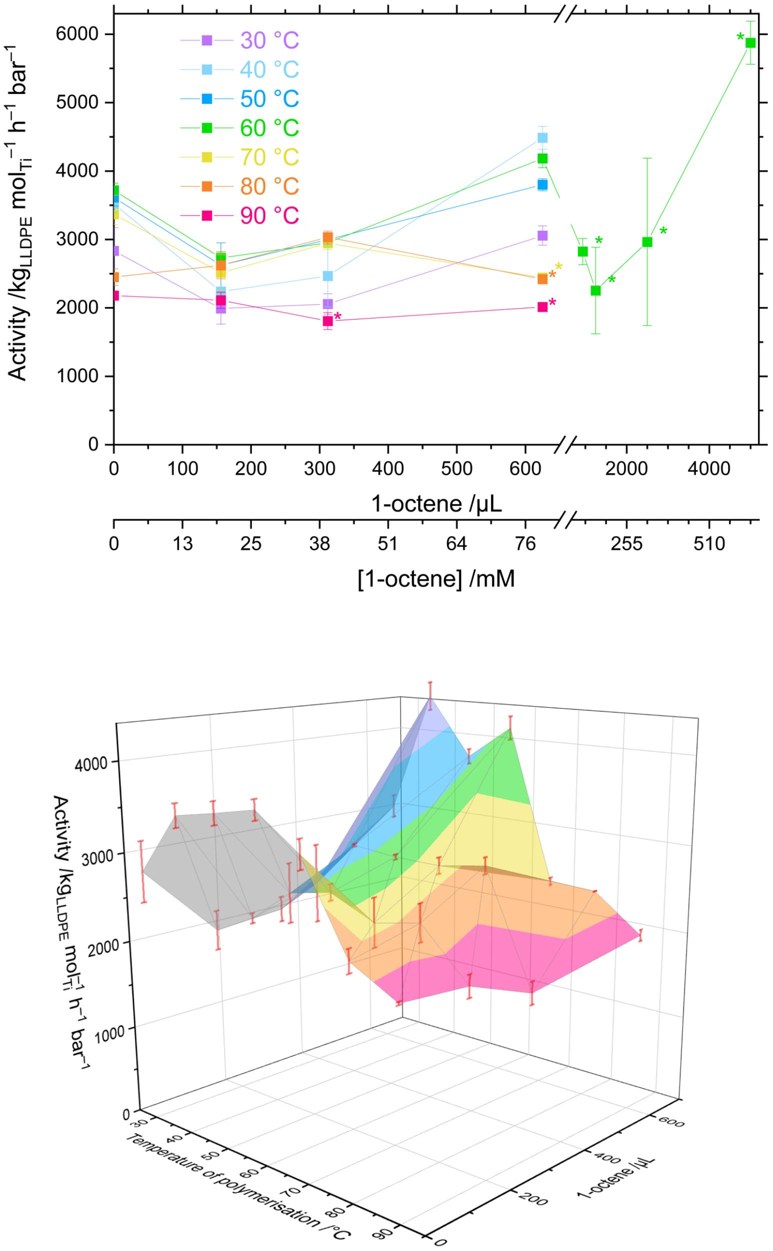

Figure S4 Mean copolymerisation activity of **1**/TIBA as a function of temperature of polymerisation (30 ≤ T_p ≤ 90 °C) and 1-octene concentration (0 ≤ V ≤ 5000 µL; 0 ≤ c ≤ 637 mM). Polymerisation conditions: 10 mg catalyst, 2 bar ethylene, 50 mL hexanes (total volume), 150 mg TIBA, and 30 minutes. Error bars shown at one standard deviation. Asterisk (*) denotes gel formation.

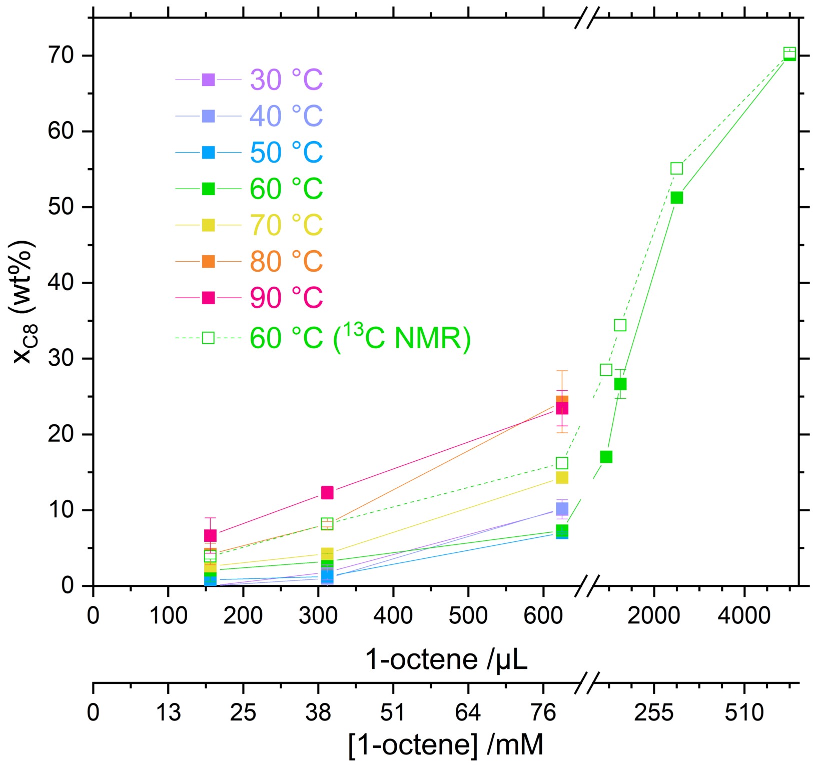

Figure S5 Mean comonomer incorporation, as a function of concentration of 1-octene, of LLDPE-C8 synthesised by **1**/TIBA, obtained from GPC-IR or ^{13}C NMR spectroscopic measurements. Error bars shown at one standard deviation.

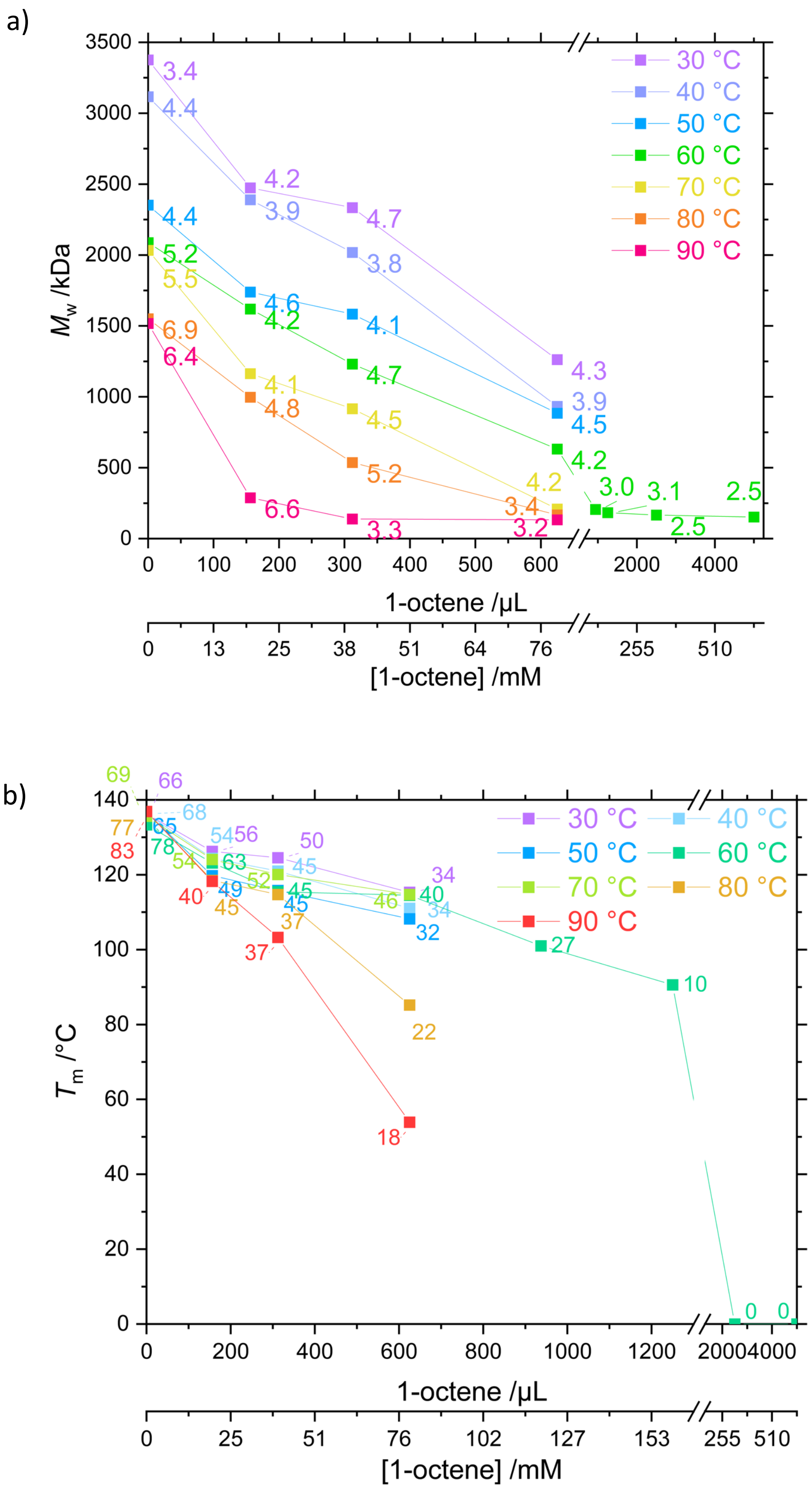

Figure S6 a) Weight-average molecular weight (with PDIs annotated) of LLDPE-C8 synthesised by **1**/TIBA as a function of temperature of polymerisation and 1-octene concentration. b) Melting temperature (with crystallinity annotated) of LLDPE-C8 synthesised by **1**/TIBA as a function of temperature of polymerisation and comonomer concentration.

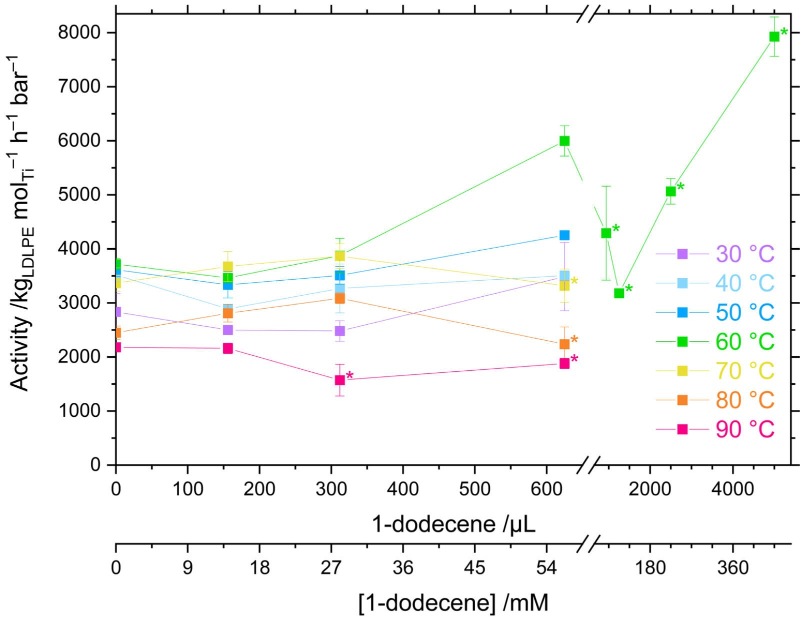

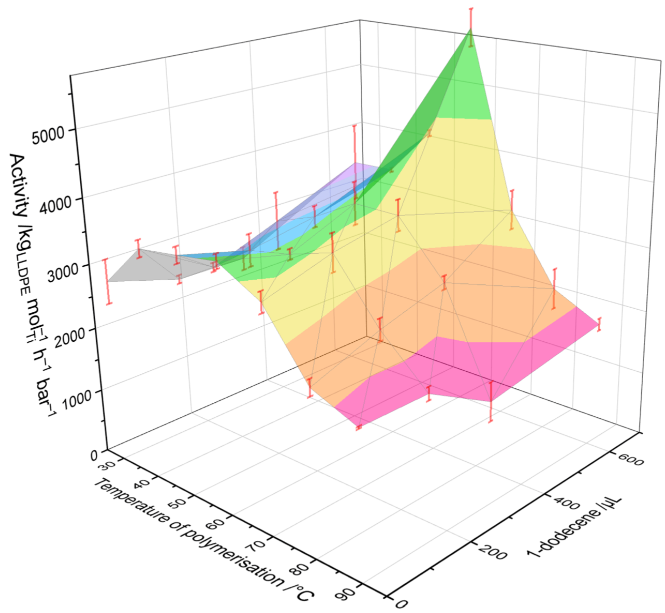

Figure S7 Mean copolymerisation activity of **1**/TIBA as a function of temperature of polymerisation (30 ≤ T_p ≤ 90 °C) and 1-dodecene concentration (0 ≤ V ≤ 5000 µL; 0 ≤ c ≤ 451 mM). Polymerisation conditions: 10 mg catalyst, 2 bar ethylene, 50 mL hexanes (total volume), 150 mg TIBA, and 30 minutes. Error bars shown at one standard deviation. Asterisk (*) denotes gel formation.

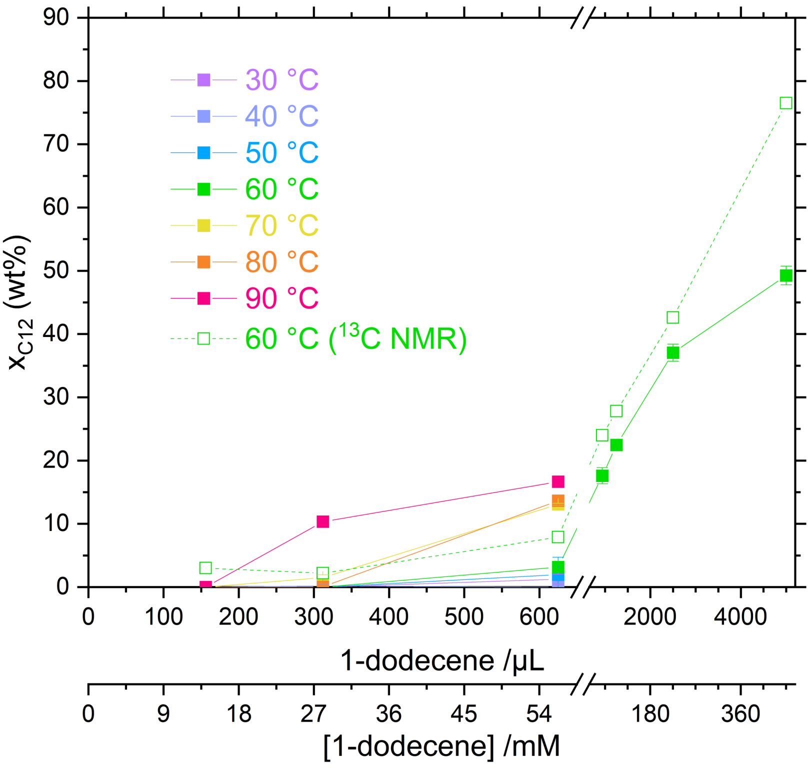

Figure S8 Mean comonomer incorporation, as a function of concentration of 1-dodecene, of LLDPE-C12 synthesised by **1**/TIBA, obtained from GPC-IR or ^{13}C NMR spectroscopic measurements. Error bars shown at one standard deviation.

a)

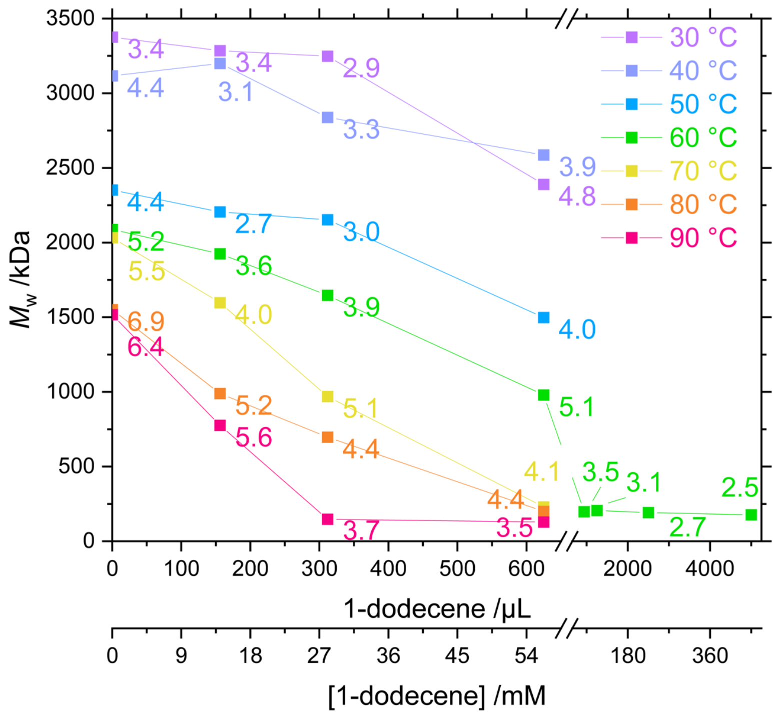

b)

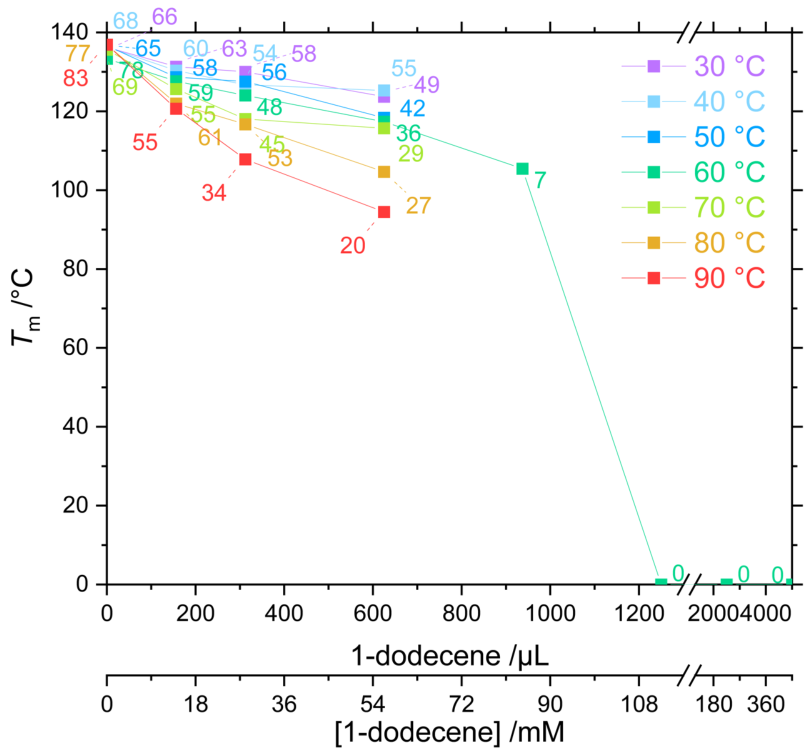

Figure S9 a) Weight-average molecular weight (with PDIs annotated) of LLDPE-C12 synthesised by **1**/TIBA as a function of temperature of polymerisation and 1-dodecene concentration. b) Melting temperature (with crystallinity annotated) of LLDPE-C12 synthesised by **1**/TIBA as a function of temperature of polymerisation and comonomer concentration.

6. Gel-permeation chromatography

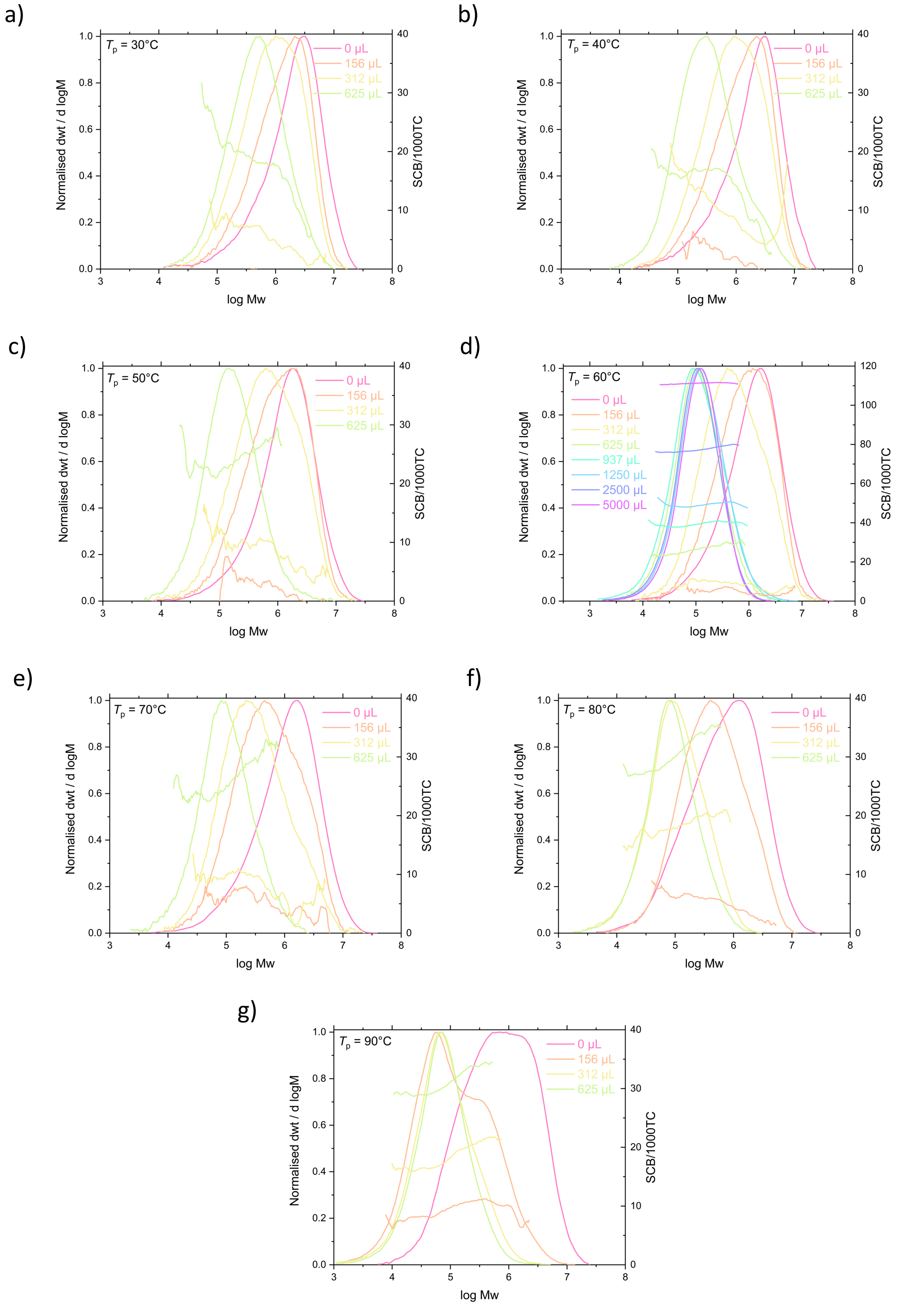

Figure S10 Gel permeation chromatograms and SCB/1000TC of LLDPE-C6 synthesised by **1**/TIBA as a function of comonomer loading. Polymerisation conditions: 10 mg catalyst, 150 mg TIBA, 2 bar ethylene, 0–5 mL 1-hexene, 50 mL hexanes, 30 minutes, and T_p = 30 (a), 40 (b), 50 (c), 60 (d), 70 (e), 80 (f) or 90 (g) °C.

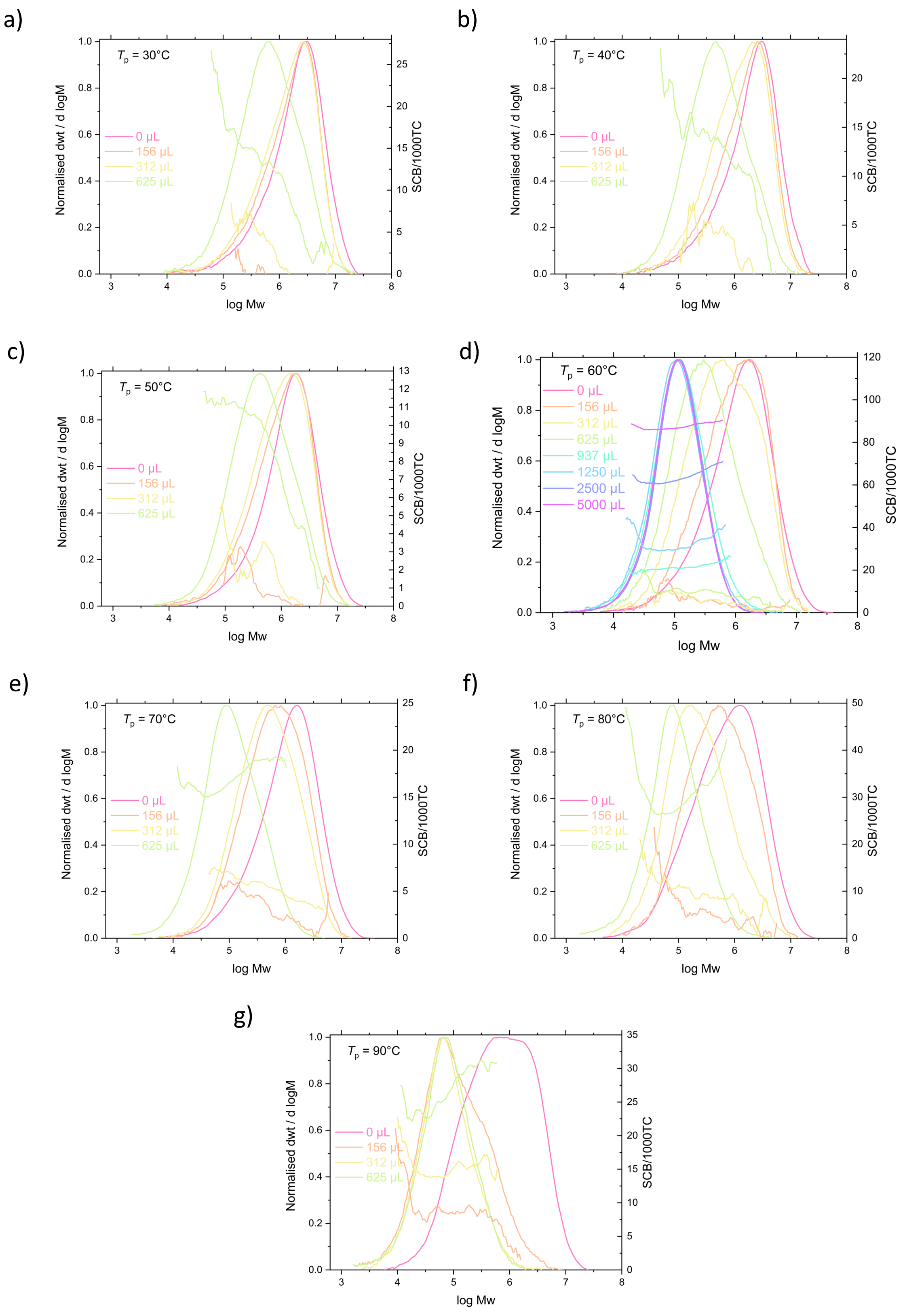

Figure S11 Gel permeation chromatograms and SCB/1000TC of LLDPE-C8 synthesised by **1**/TIBA as a function of comonomer loading. Polymerisation conditions: 10 mg catalyst, 150 mg TIBA, 2 bar ethylene, 0–5 mL 1-octene, 50 mL hexanes, 30 minutes, and T_p = 30 (a), 40 (b), 50 (c), 60 (d), 70 (e), 80 (f) or 90 (g) °C.

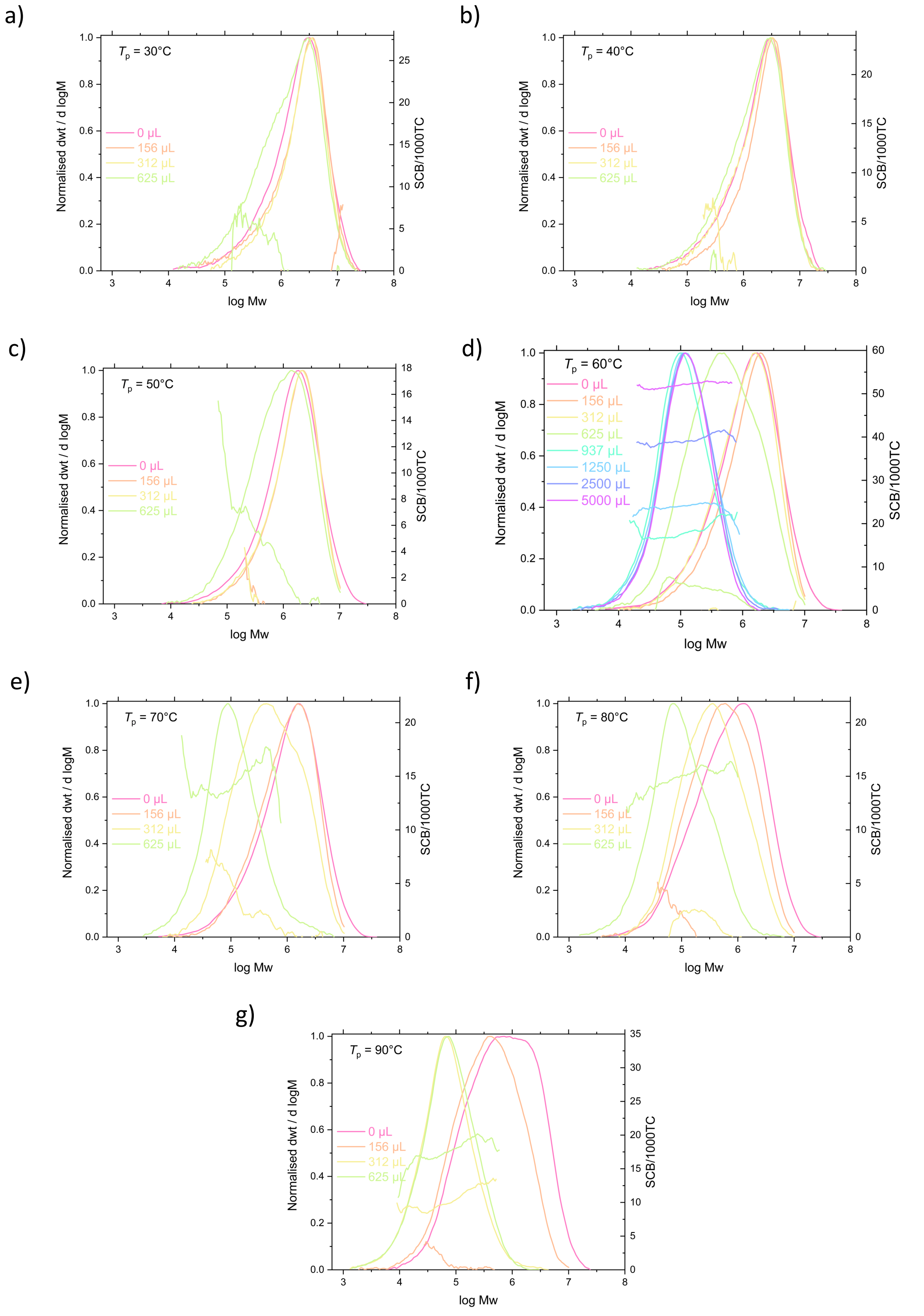

Figure S12 Gel permeation chromatograms and SCB/1000TC of LLDPE-C12 synthesised by **1**/TIBA as a function of comonomer loading. Polymerisation conditions: 10 mg catalyst, 150 mg TIBA, 2 bar ethylene, 0–5 mL 1-dodecene, 50 mL hexanes, 30 minutes, and T_p = 30 (a), 40 (b), 50 (c), 60 (d), 70 (e), 80 (f) or 90 (g) °C.

7. Differential scanning calorimetry

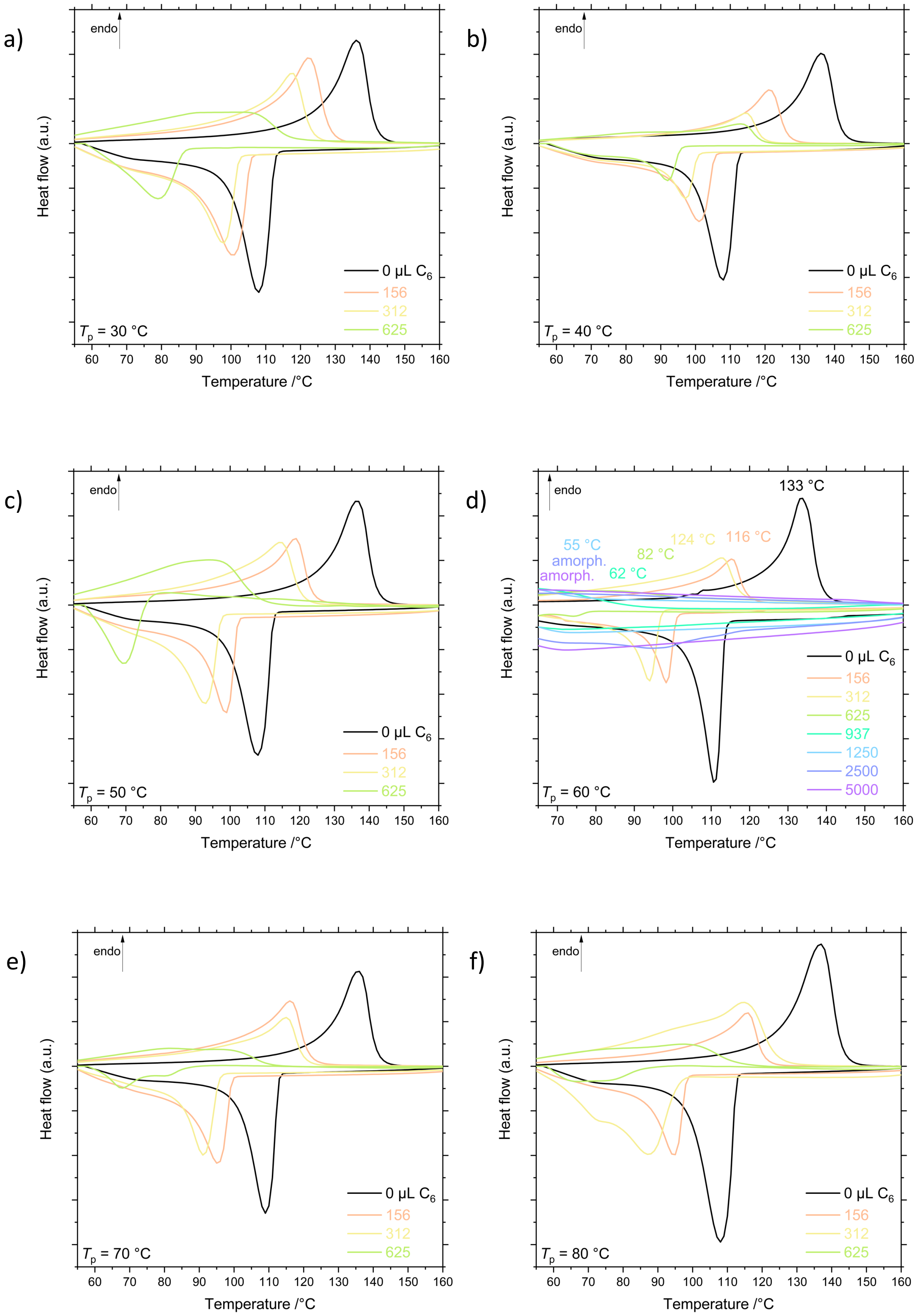

g)

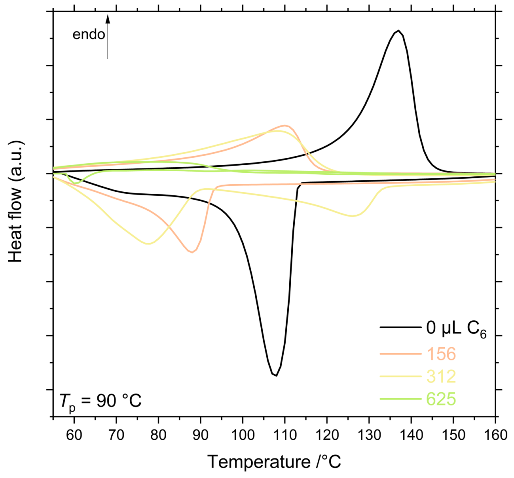

Figure S13 Differential scanning calorimetry (20 K min^{-1}, second cycle plotted) of LLDPE-C6 synthesised by **1**/TIBA as a function of comonomer loading. Polymerisation conditions: 10 mg catalyst, 150 mg TIBA, 2 bar ethylene, 0–5 mL 1-hexene, 50 mL hexanes, 30 minutes, and T_p = 30 (a), 40 (b), 50 (c), 60 (d), 70 (e), 80 (f) or 90 (g) °C.

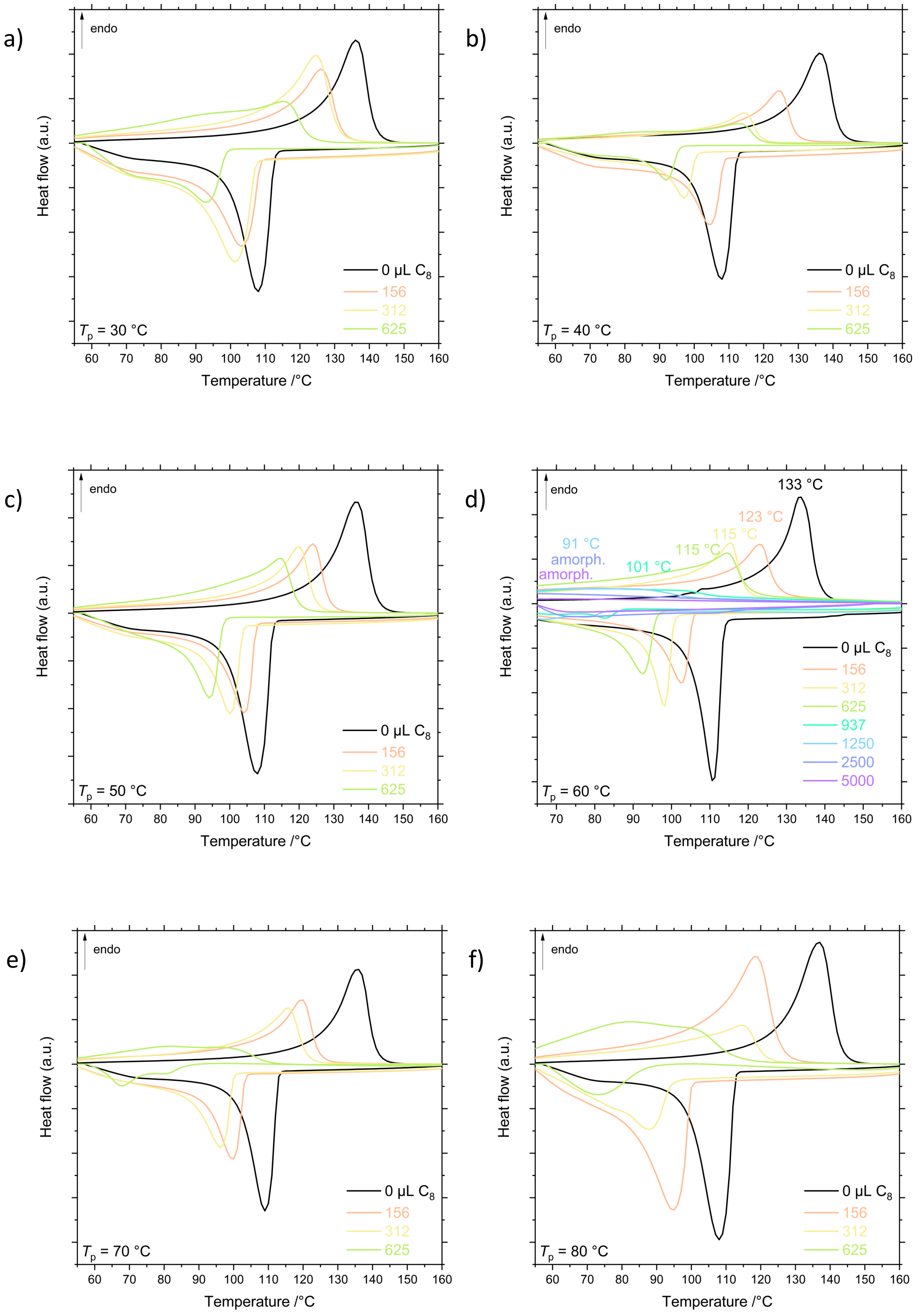

a)
endo
Heat flow (a.u.)
0 µL C8
156
312
625
Tp = 30 °C
Temperature /°C
b)
endo
Heat flow (a.u.)
0 µL C8
156
312
625
Tp = 40 °C
Temperature /°C
c)
endo
Heat flow (a.u.)
0 µL C8
156
312
625
Tp = 50 °C
Temperature /°C
d)
endo
133 °C
123 °C
115 °C
115 °C
91 °C
amorph.
amorph.
101 °C
Heat flow (a.u.)
0 µL C8
156
312
625
937
1250
2500
5000
Tp = 60 °C
Temperature /°C
e)
endo
Heat flow (a.u.)
0 µL C8
156
312
625
Tp = 70 °C
Temperature /°C
f)
endo
Heat flow (a.u.)
0 µL C8
156
312
625
Tp = 80 °C
Temperature /°C

g)

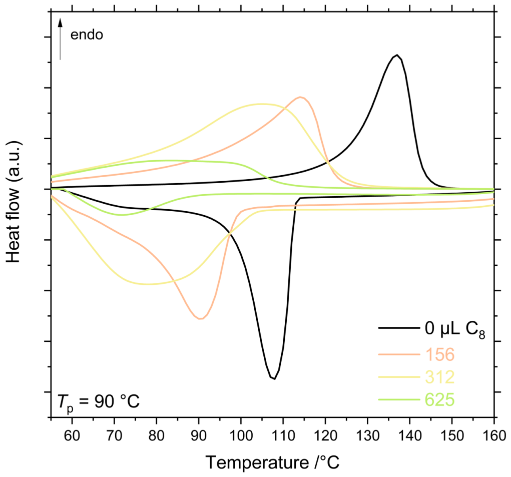

Figure S14 Differential scanning calorimetry (20 K min^{-1}, second cycle plotted) of LLDPE-C8 synthesised by **1**/TIBA as a function of comonomer loading. Polymerisation conditions: 10 mg catalyst, 150 mg TIBA, 2 bar ethylene, 0–5 mL 1-octene, 50 mL hexanes, 30 minutes, and and T_p = 30 (a), 40 (b), 50 (c), 60 (d), 70 (e), 80 (f) or 90 (g) °C.

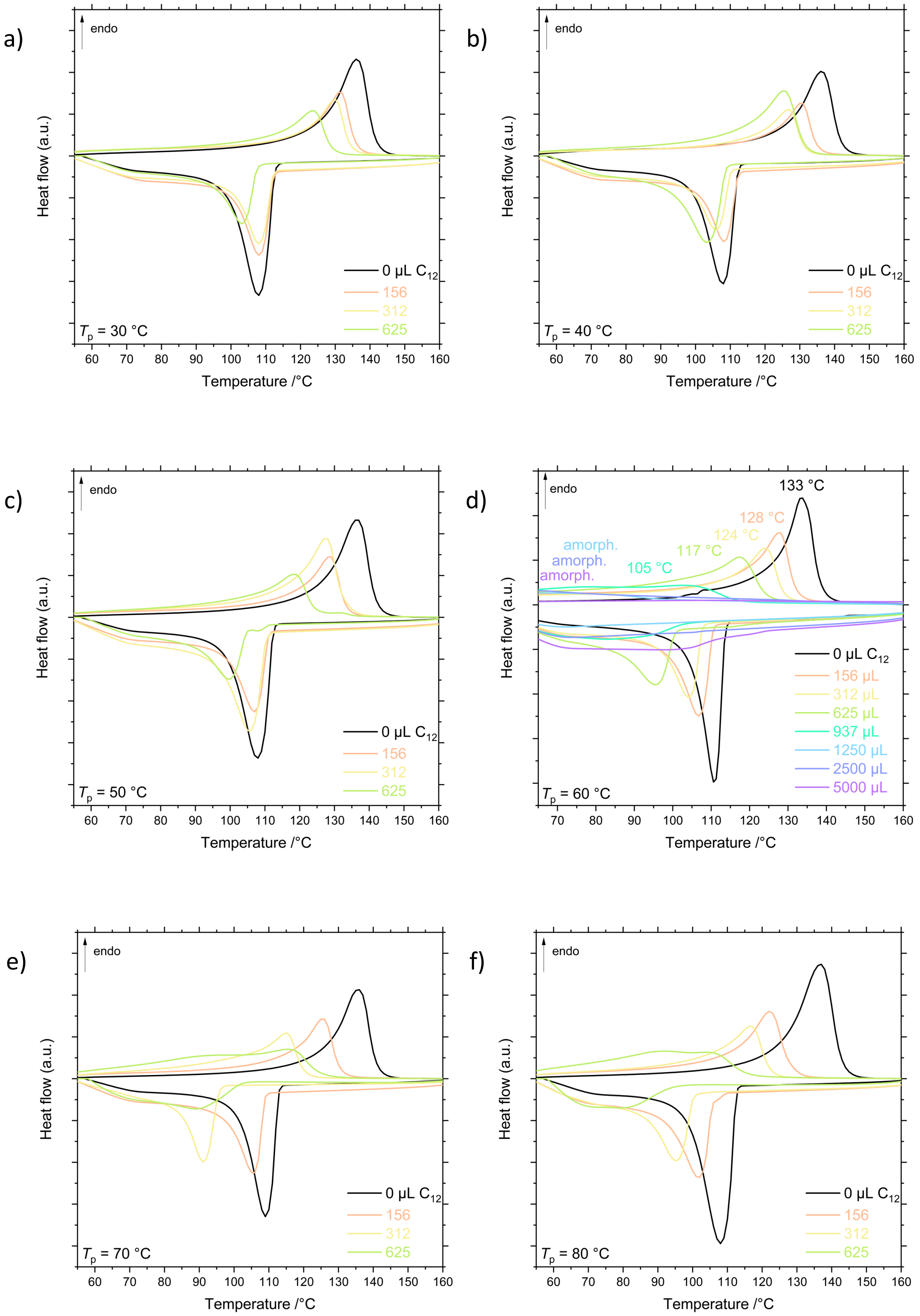

a)
endo
Heat flow (a.u.)
Temperature /°C
0 µL C_{12}
156
312
625
T_p = 30 °C
b)
T_p = 40 °C
c)
T_p = 50 °C
d)
133 °C
128 °C
124 °C
117 °C
105 °C
amorph.
amorph.
amorph.
156 µL
312 µL
625 µL
937 µL
1250 µL
2500 µL
5000 µL
T_p = 60 °C
e)
T_p = 70 °C
f)
T_p = 80 °C

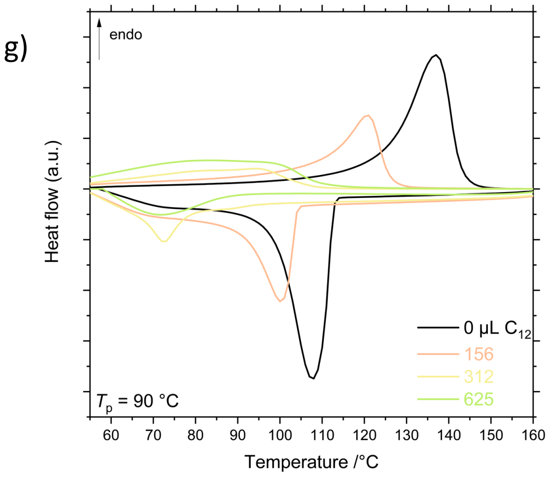

Figure S15 Differential scanning calorimetry (20 K min^{-1}, second cycle plotted) of LLDPE-C12 synthesised by **1**/TIBA as a function of comonomer loading. Polymerisation conditions: 10 mg catalyst, 150 mg TIBA, 2 bar ethylene, 0–5 mL 1-dodecene, 50 mL hexanes, 30 minutes, and T_p = 30 (a), 40 (b), 50 (c), 60 (d), 70 (e), 80 (f) or 90 (g) °C.

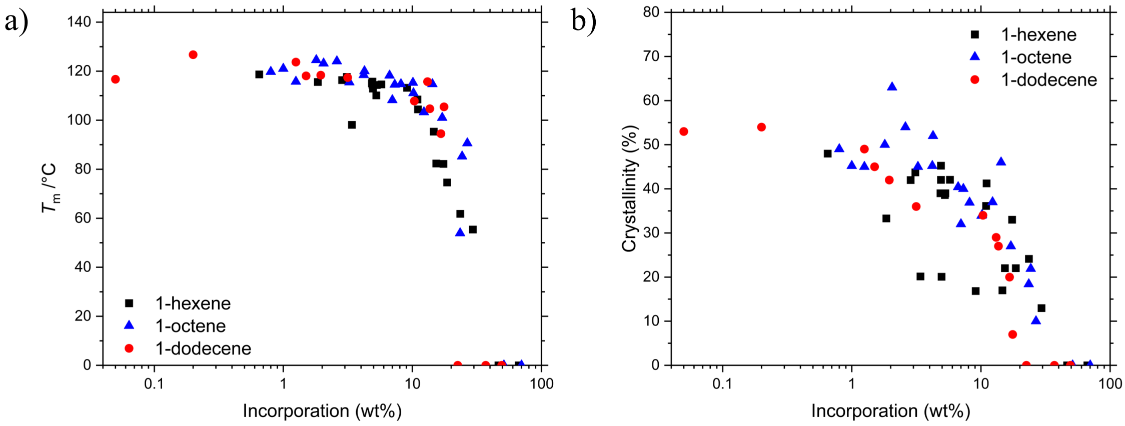

Figure S16 a) Melting point and b) crystallinity of LLDPE as a function of comonomer incorporation (measured by GPC–IR). Synthesised by **1**/TIBA using C6, C8, and C12 at 30 ≤ T_p ≤ 90 °C and 156–5000 µL LAO.

8. Rheology

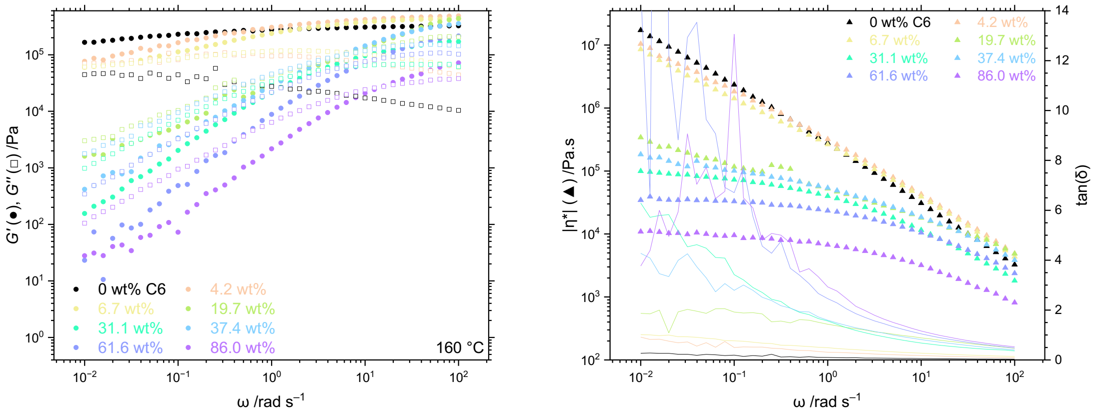

Figure S17 Frequency sweep rheometric curves measured at 160 °C for LLDPE-C6 synthesised by **1**/TIBA. Storage modulus, *G*′; loss modulus, *G*′′; complex viscosity, |η*|; phase angle, δ; angular frequency, ω. Polymerisation conditions: 10 mg catalyst, 150 mg TIBA, 2 bar ethylene, 0–5 mL 1-hexene, 50 mL hexanes, 30 minutes, and 60 °C.

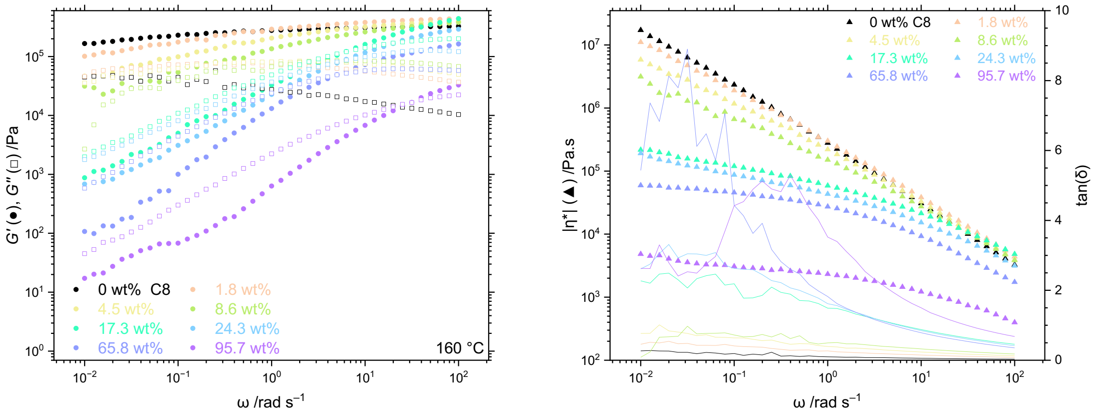

Figure S18 Frequency sweep rheometric curves measured at 160 °C for LLDPE-C8 synthesised by **1**/TIBA. Storage modulus, *G*′; loss modulus, *G*′′; complex viscosity, |η*|; phase angle, δ; angular frequency, ω. Polymerisation conditions: 10 mg catalyst, 150 mg TIBA, 2 bar ethylene, 0–5 mL 1-octene, 50 mL hexanes, 30 minutes, and 60 °C.

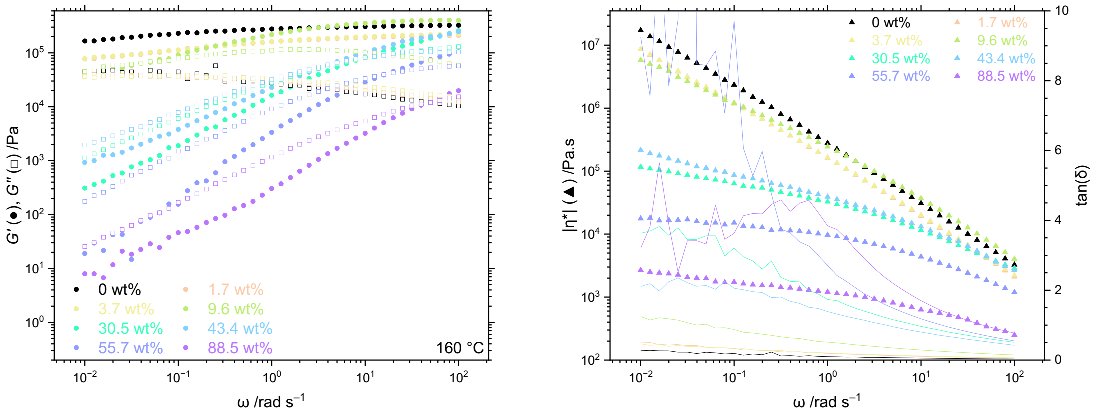

Figure S19 Frequency sweep rheometric curves measured at 160 °C for LLDPE-C12 synthesised by **1**/TIBA. Storage modulus, *G*′; loss modulus, *G*′′; complex viscosity, |η*|; phase angle, δ; angular frequency, ω. Polymerisation conditions: 10 mg catalyst, 150 mg TIBA, 2 bar ethylene, 0–5 mL 1-dodecene, 50 mL hexanes, 30 minutes, and 60 °C.

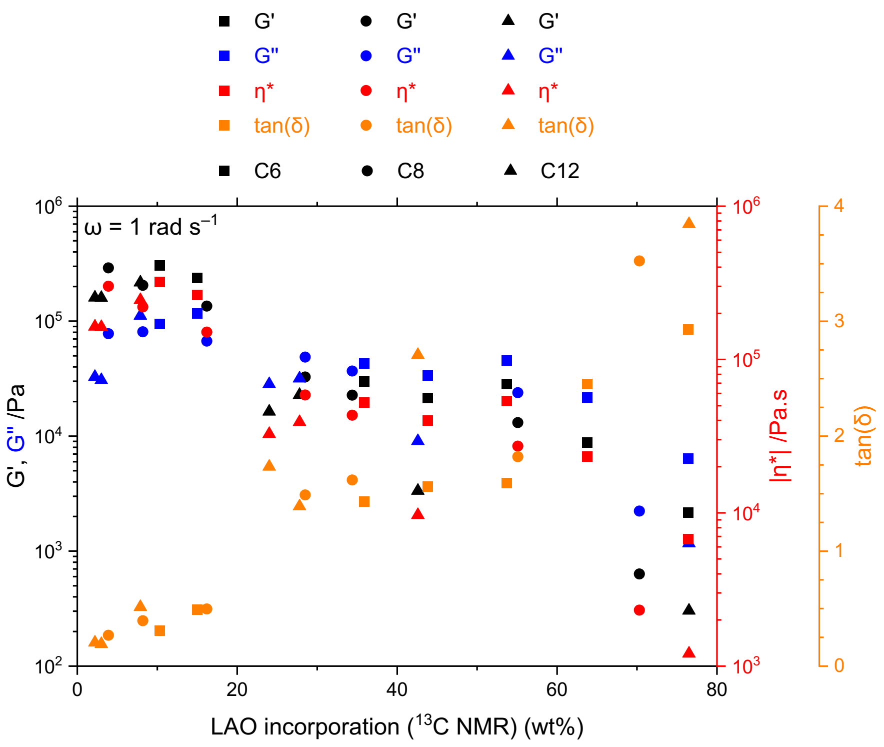

Figure S20 Rheological parameters (*G*′, *G*′′, |η*|, tan(δ); measured at 160 °C and ω = 1.0 rad s^{-1}) of LLDPE as a function of comonomer incorporation (measured by ^{13}C NMR spectroscopy). Synthesised by **1**/TIBA using C6, C8, and C12 at 30 ≤ T_p ≤ 90 °C and 156–5000 µL LAO.

9. NMR spectroscopy

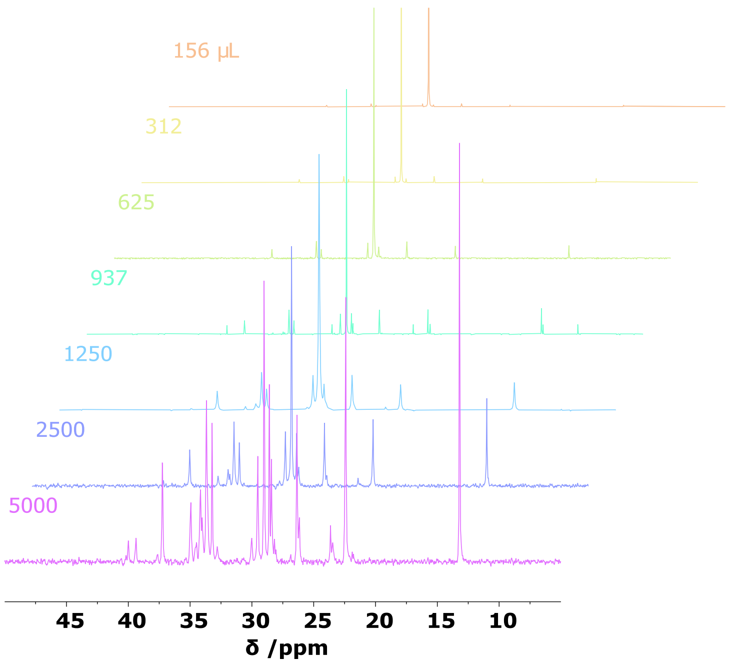

Figure S21 Quantitative ^{13}C{^{1}H} NMR spectra (151 MHz, 1,1,2,2-tetrachloroethane-d_2, 403 K, 50 ≥ δ ≥ 5 ppm) of LLDPE-C6 synthesised by **1**/TIBA at 60 °C.

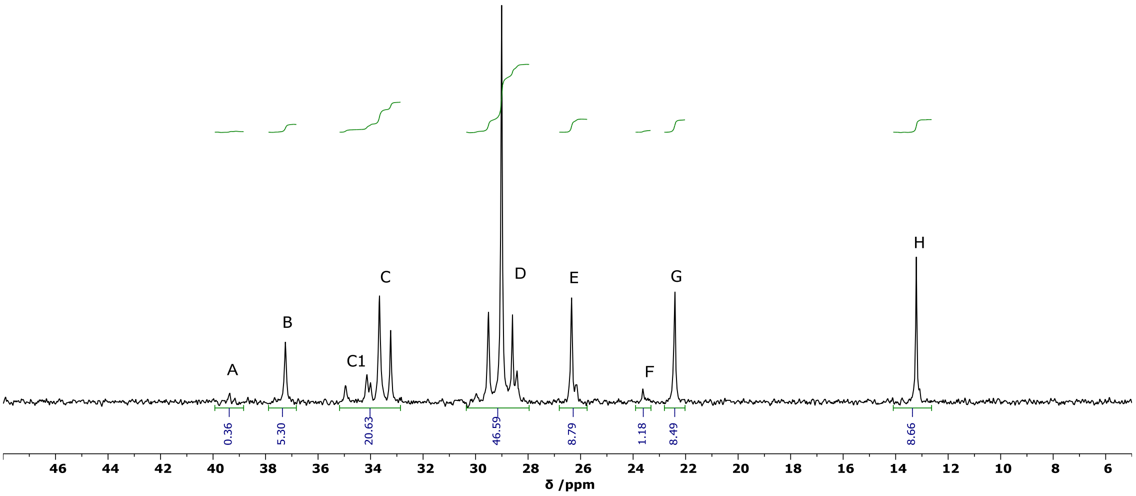

Figure S22 Quantitative $^{13}C\{^{1}H\}$ NMR spectrum (151 MHz, 1,1,2,2-tetrachloroethane-d_2, 403 K, 48 ≥ δ ≥ 5 ppm) of LLDPE-C6, 37.0 mol% incorporation. Integration according to Galland *et al.*.[9]

[HHH] = 2A + B – G

[EHH] = C_1

[EHE] = B

[EEE] = ½ D – ½ G – ¼ E

[HEH] = F

[HEE] = E

Table S3 Triad distributions of LLDPE-C6, synthesised at T_p = 60 °C, obtained from ^{13}C NMR spectroscopy.

	mole fraction		triad distribution					
C6 /µL	[E]	[H]	[HHH]	[EHH]	[EHE]	[EEE]	[HEH]	[HEE]
156	0.96	0.04	0.00	0.04	0.01	0.93	0.00	0.02
312	0.94	0.06	–0.01	0.06	0.02	0.89	0.00	0.03
625	0.84	0.16	–0.01	0.16	0.05	0.71	0.00	0.10
937	0.79	0.21	–0.04	0.22	0.07	0.59	0.03	0.13
1250	0.72	0.28	0.00	0.27	0.07	0.51	0.01	0.14
2500	0.63	0.37	–0.04	0.40	0.10	0.33	0.02	0.18
5000	0.48	0.52	–0.04	0.59	0.10	0.14	0.05	0.17

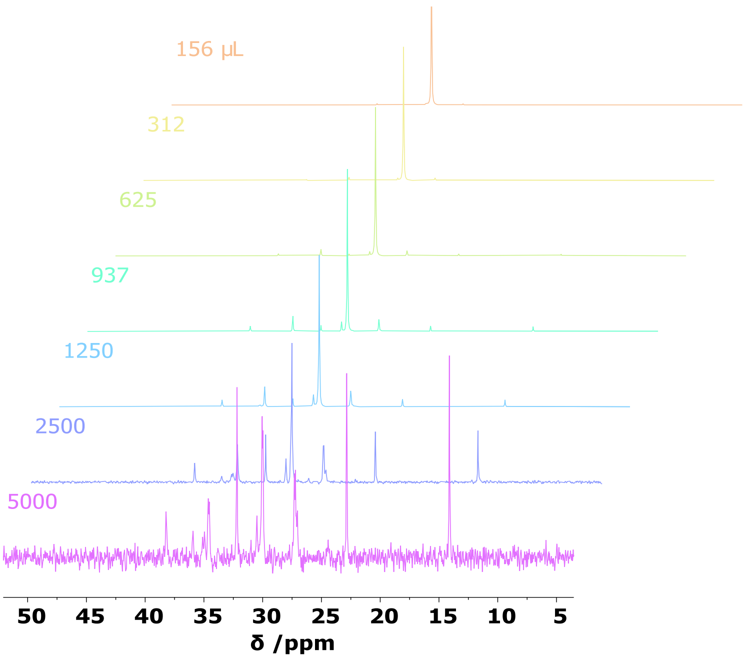

Figure S23 Quantitative $^{13}C\{^{1}H\}$ NMR spectra (151 MHz, 1,1,2,2-tetrachloroethane-d_2, 403 K, 50 ≥ δ ≥ 5 ppm) of LLDPE-C8 synthesised by **1**/TIBA at 60 °C.

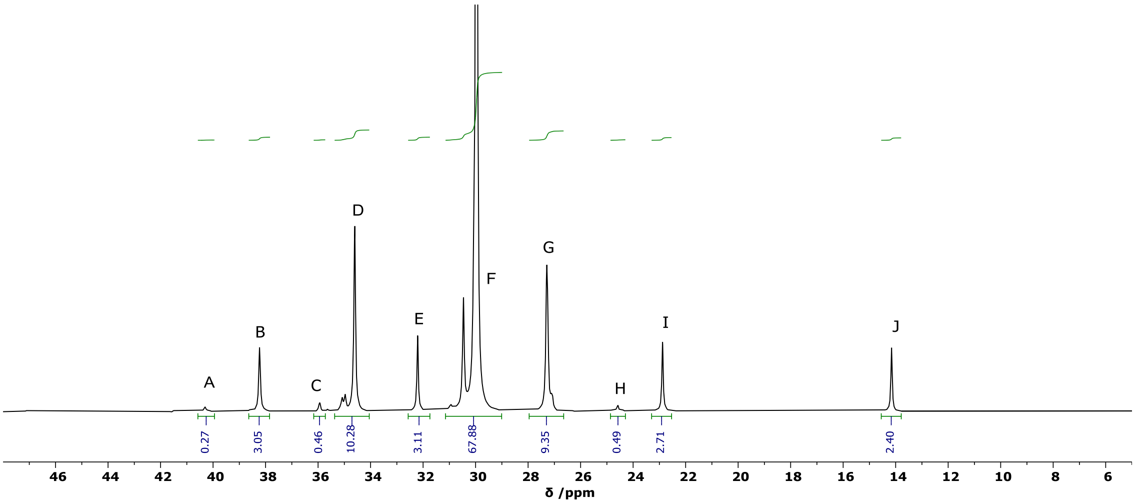

Figure S24 Quantitative $^{13}C\{^{1}H\}$ NMR spectrum (128 MHz, 1,1,2,2-tetrachloroethane-d_2, 403 K, 48 ≥ δ ≥ 5 ppm) of LLDPE-C8, 11.6 mol% incorporation. Integration according to Galland *et al.*.[9]

[OOO] = A – ½ C

[EOO] = C

[EOE] = B

[EEE] = ½ F – ¼ E – ¼ G

[OEO] = H

[OEE] = G – E

Table S4 Triad distributions of LLDPE-C8, synthesised at T_p = 60 °C, obtained from ^{13}C NMR spectroscopy.

	mole fraction		triad distribution					
C8 /µL	[E]	[O]	[OOO]	[EOO]	[EOE]	[EEE]	[OEO]	[OEE]
156	0.99	0.01	0.01	0.00	0.00	0.98	0.00	0.02
312	0.98	0.02	0.01	0.00	0.00	0.95	0.00	0.03
625	0.95	0.05	0.03	0.01	0.00	0.91	0.00	0.04
937	0.91	0.09	0.05	0.01	0.00	0.82	0.00	0.12
1250	0.88	0.12	0.07	0.00	0.00	0.76	0.01	0.16
2500	0.77	0.23	0.14	0.03	0.01	0.54	0.00	0.29
5000	0.63	0.37	0.30	–0.03	–0.02	0.37	0.05	0.33

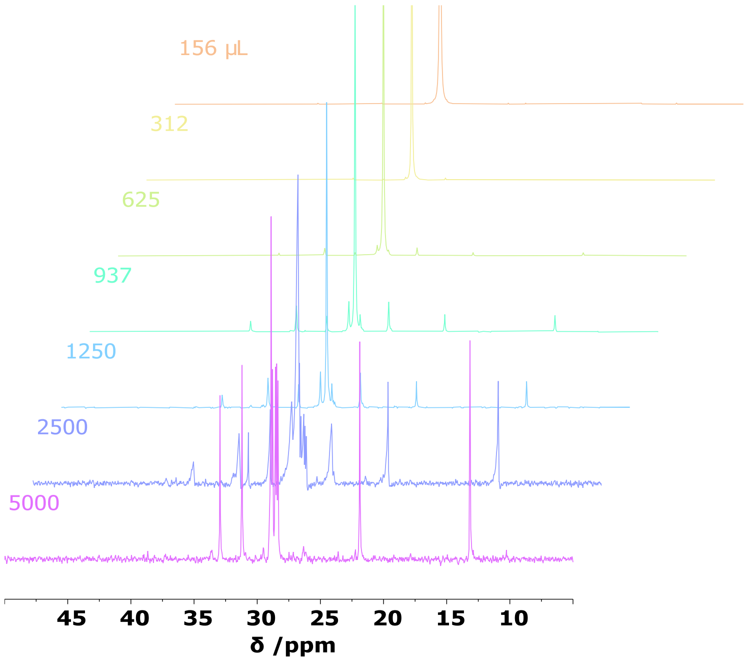

Figure S25 Quantitative $^{13}C\{^1H\}$ NMR spectra (151 MHz, 1,1,2,2-tetrachloroethane-d_2, 403 K, 50 ≥ δ ≥ 5 ppm) of LLDPE-C12 synthesised by **1**/TIBA at 60 °C.

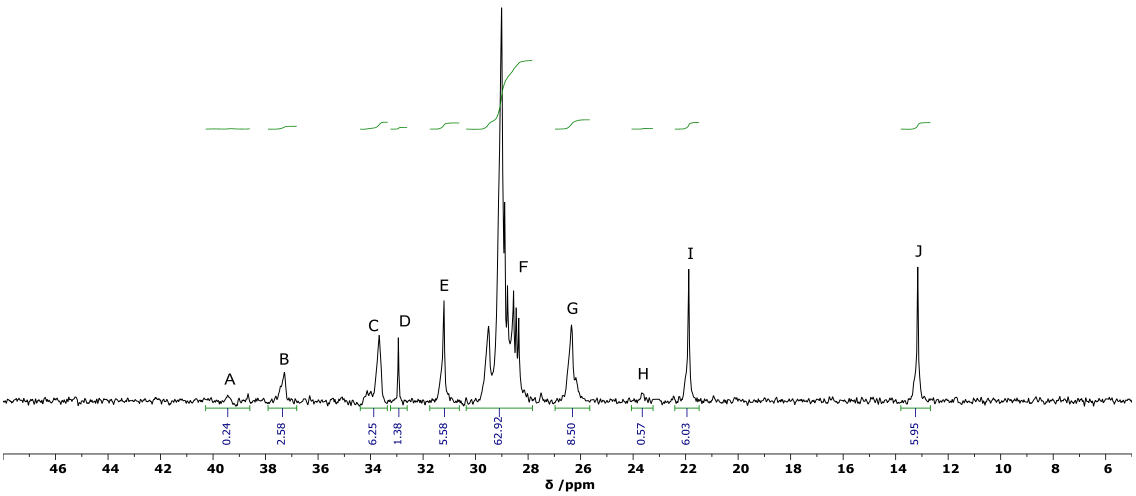

Figure S26 Quantitative $^{13}C\{^1H\}$ NMR spectrum (151 MHz, 1,1,2,2-tetrachloroethane-d_2, 403 K, 48 ≥ δ ≥ 5 ppm) of LLDPE-C12, 11.0 mol% incorporation. Integration based on Galland *et al.*.[9]

[DDD] = A – ½ C

[EDD] = C

[EDE] = B

[EEE] = ½ F – ½ E – ½ G – I

[DED] = H

[DEE] = G – I

Table S5 Diad distributions of LLDPE-C12, synthesised at T_p = 60 °C, obtained from ^{13}C NMR spectroscopy.

	mole fraction		diad distribution		
C12 /µL	[E]	[D]	[EE]	[ED]	[DD]
156	0.995	0.005	0.992	0.005	0.003
312	0.996	0.004	0.992	0.010	–0.001
625	0.986	0.014	0.972	0.027	0.001
937	0.950	0.050	0.904	0.093	0.004
1250	0.940	0.060	0.880	0.119	0.001
2500	0.890	0.110	0.787	0.207	0.006
5000	0.648	0.352	0.298	0.701	0.002

Where comonomer C = H, O, or D:

[EE] = [EEE] + ½ [CEE]

[EC] = [CEC] + ½ [CEE] + [ECE] + ½ [ECC]

[CC] = [CCC] + ½ [ECC]

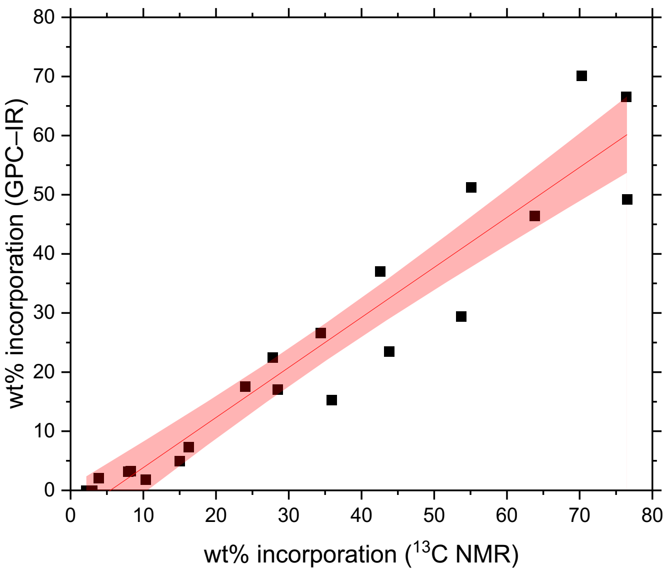

Figure S27 Correlation between LAO incorporation determined from quantitative high-temperature $^{13}C\{^1H\}$ NMR spectroscopy and from GPC–IR. Regression statistics: y = (0.847 ± 0.285) x + (–0.0462 ± 0.117); R^2 = 0.9072.

Conversions between wt% and mol%:

$$x_{C,wt\%} = \frac{w_C}{w_E + w_C} = \frac{n_C M_{r,C}}{n_E M_{r,E} + n_C M_{r,C}} = \frac{x_{C,mol\%} M_{r,C}}{\left(1 - x_{C,mol\%}\right) M_{r,E} + x_{C,mol\%} M_{r,C}}$$

$$x_{C,mol\%} = \frac{n_C}{n_E + n_C} = \frac{w_C / M_{r,C}}{w_E / M_{r,E} + w_C / M_{r,C}} = \frac{x_{C,wt\%} / M_{r,C}}{\left(1 - x_{C,wt\%}\right) / M_{r,E} + x_{C,wt\%} / M_{r,C}}$$

10. Fineman-Ross analysis

$$f = F \frac{r_1 F + 1}{r_2 + F} \Rightarrow \frac{F}{f}(f - 1) = r_1 \frac{F^2}{f} - r_2$$

$$r_1 = \frac{k_{11}}{k_{12}};\ r_2 = \frac{k_{22}}{k_{21}}$$

where F = [E]/[LAO] in feed, f = [E]/[LAO] in copolymer, and k_{nm} is the rate constant for the insertion of monomer m after monomer n.

Table S6 Reactivity ratios for E/LAO copolymerisations at T_p = 60 °C.

Copolymerisation	r_E	r_{LAO}	$r_E * r_{LAO}$
E/C6	10.2 ± 0.6	0.26 ± 0.06	2.66 ± 0.63
E/C8	17.4 ± 4.8	0.73 ± 0.42	12.72 ± 8.12
E/C12	13.4 ± 0.5	0.03 ± 0.05	0.42 ± 0.70

Table S7 Fineman-Ross data for E/C6 copolymerisation at T_p = 60 °C. F = [E]/[LAO] in feed, f = [E]/[LAO] (= $\frac{1}{x_{LAO}} - 1$) in copolymer. [E] calculated from Kissin's equation, $[E] \approx p \cdot 0.00175 \cdot exp\left(\frac{2569}{1.98 \cdot T}\right)$ for partial pressure, *p*, in bar and absolute temperature *T*.

C6 /µL	[E] /mM	[C6] /mM	x_{C6} (mol%)	F	f
156	172	24.9	0.6	6.89	159.2
312	172	49.9	1.7	3.45	57.6
625	172	100.0	5.7	1.72	16.6
937	172	149.9	9.3	1.15	9.8
1250	172	199.9	12.2	0.86	7.2
2500	172	399.8	22.4	0.43	3.5
5000	172	799.7	39.9	0.22	1.5

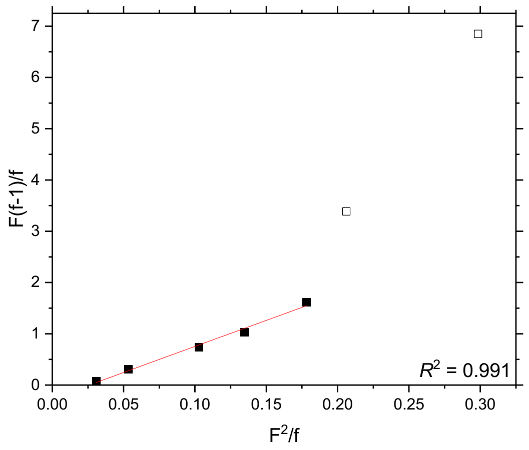

Figure S28 Fineman-Ross plot for ethylene/1-hexene copolymerisation at T_p = 60 °C. Filled squares denote soluble LLDPE.

Table S8 Fineman-Ross data for E/C8 copolymerisation at T_p = 60 °C. F = [E]/[LAO] in feed, f = [E]/[LAO] (= $\frac{1}{x_{LAO}} - 1$) in copolymer. [E] calculated from Kissin's equation, $[E] \approx p \cdot 0.00175 \cdot exp\left(\frac{2569}{1.98 \cdot T}\right)$ for partial pressure, *p*, in bar and absolute temperature *T*.

C8 /µL	[E] /mM	[C8] /mM	x_{C8} (mol%)	F	f
156	172	19.9	0.5	8.65	191.2
312	172	39.8	0.8	4.32	119.1
625	172	79.6	1.9	2.16	50.8
937	172	119.4	4.9	1.44	19.5
1250	172	159.3	8.3	1.08	11.0
2500	172	318.5	20.8	0.54	3.8
5000	172	637.0	36.9	0.27	1.7

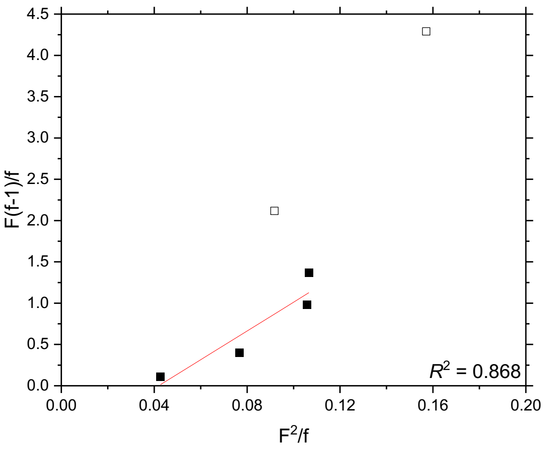

Figure S29 Fineman-Ross plot for ethylene/1-octene copolymerisation at T_p = 60 °C. Filled squares denote soluble LLDPE.

Table S9 Fineman-Ross data for E/C12 copolymerisation at T_p = 60 °C. F = [E]/[LAO] in feed, f = [E]/[LAO] (= $\frac{1}{x_{LAO}} - 1$) in copolymer. [E] calculated from Kissin's equation, $[E] \approx p \cdot 0.00175 \cdot exp\left(\frac{2569}{1.98 \cdot T}\right)$ for partial pressure, *p*, in bar and absolute temperature *T*.

C12 /µL	[E] /mM	[C12] /mM	x_{C12} (mol%)	F	f
156	172	14.1	0.00	12.23	-
312	172	28.1	0.00	6.12	-
625	172	56.3	0.54	3.05	184.5
937	172	84.4	3.44	2.04	28.1
1250	172	112.6	4.60	1.53	20.7
2500	172	225.3	8.93	0.76	10.2
5000	172	450.6	13.92	0.38	6.2

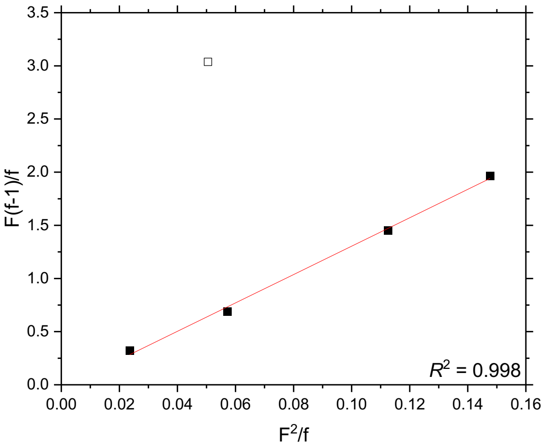

Figure S30 Fineman-Ross plot for ethylene/1-dodecene copolymerisation at T_p = 60 °C. Filled squares denote soluble LLDPE.

11. Multiple linear regression

When attempting to analyse the various LAO copolymerisation systems simultaneously, all of the relevant variables must first be identified. The explanatory variables are identified as the temperature of polymerisation (T_p), the identity of the comonomer (enumerated as the number of carbon atoms in the LAO, n), and the comonomer concentration (c). Other factors such as pressure, solvent, scale, scavenger, catalyst ligand, and stirring speed have been held constant in these studies but are likely to have an influence over the polymer properties. In particular, the ethylene pressure is expected to have a dramatic influence – at sufficiently high values, it is anticipated that the observed diffusion-controlled regime would be replaced by a kinetic regime in which the reactivity ratios of the catalyst to the two monomers becomes the dominating factor. The response variables are then identified as catalytic activity (A), polymer melting point (T_m), crystallinity (α), molecular weight (M_w), dispersity (PDI, Đ) and comonomer incorporation (x). Taken together, this 9-dimenstional dataset (N = 75) encompasses all of the reaction-space explored in the slurry-phase copolymerisation of ethylene with α-olefins using the PHENI* catalyst **1**/TIBA.

From an inspection of the scatterplot matrix (Figure 3), it is immediately clear that some of these factors are more strongly correlated than others. As would be expected, the physical properties of the polymers depend strongly on comonomer incorporation, which is in turn strongly correlated with comonomer concentration. While it has been shown that LAO incorporation is also influenced heavily both by the polymerisation temperature and the identity of the comonomer, the bivariate plots do not show a strong correlation, though incorporation does increase slightly with T_p. This is consistent with the interdependency of these two variables and is itself strong motivation for pursuing further multivariate analysis. Of the polymer properties, T_m shows a strong linear correlation decreasing with increasing incorporation, while α, M_w, and PDI all show decreasing but non-linear correlations.

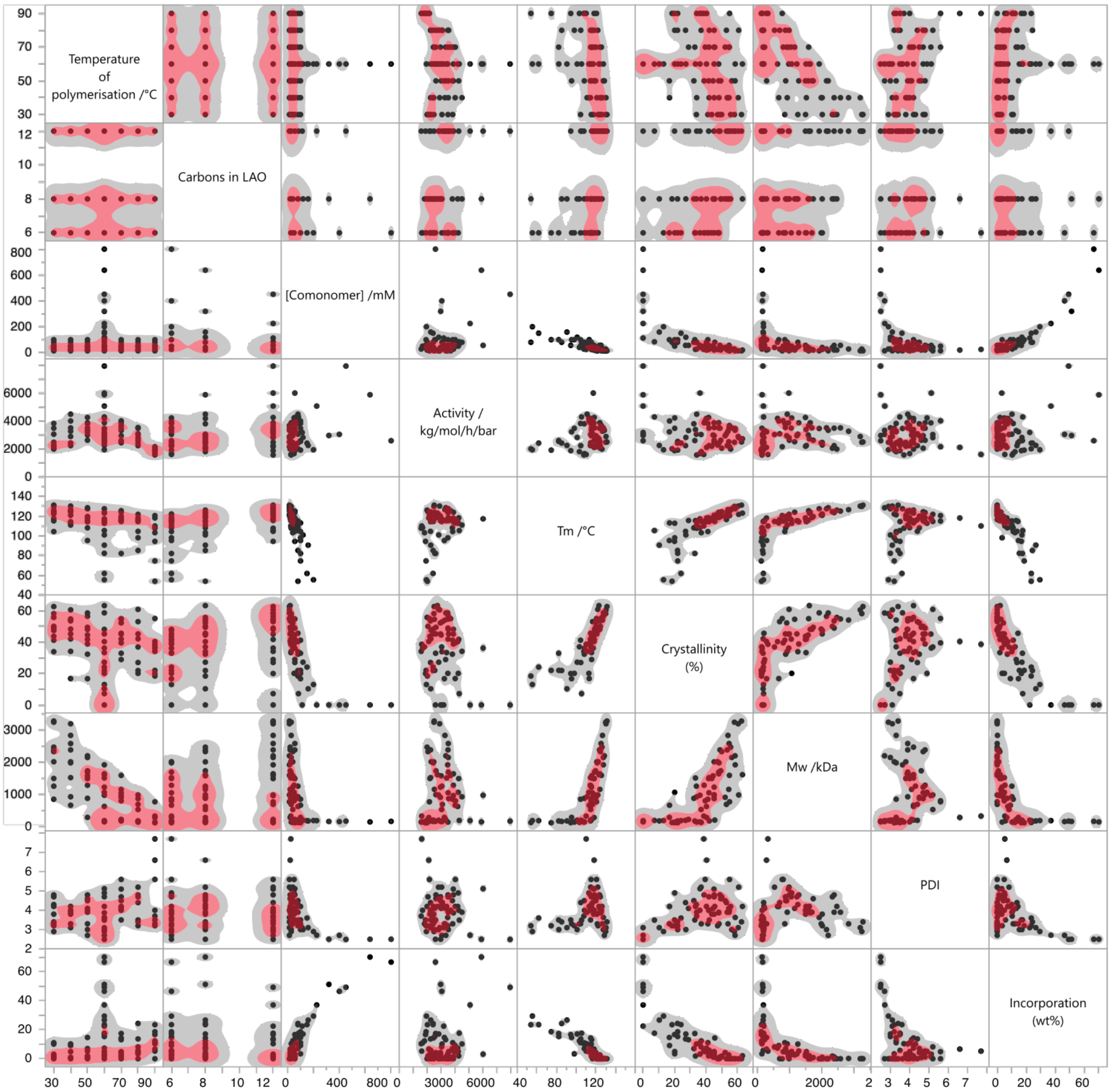

Figure S31 Scatterplot matrix of the PHENI*/E/LAO dataset, showing 2-dimensional scatter plots as functions of pairs of experimental variables. Shaded nonparametric density contours shown at the 50% and 90% quantiles. $N = 75$.

As the relationships plotted in Figure S31 are generally nonlinear, multivariable regression was performed using polynomial combinations of the explanatory variables, up to quadratic terms (T_p, c, n, T_p^2, c^2, n^2, T_pn, T_pc, cn). This resulted in a nonlinear model (model 1, M1) after least squares regression was applied to the dataset. This model allows for a detailed statistical analysis of regression, in particular the coupling of the explanatory variables to each other and the strength of their influence on the response variables. On the basis of likelihood ratio tests, T_p and c explain much of the variation of M1, with $-\log_{10}$(p-values) of 33.0 and 24.9 respectively, and all of the polynomial terms apart from n^2 have significant relationships at the .01 level of hypothesis testing. The model is predictive with R^2 values of: A (0.65), T_m (0.85), α (0.81), M_w (0.96), PDI (0.50), x (0.85). Linear regression models assume linearity, homoscedasticity, and residual normality. In this dataset, visual inspection of the distribution of the residuals, confirms that these assumptions approximately hold. Linear models are unable to fit limiting behaviour, and so the empirical lower bounds, particular of M_w, are likely to be poorly accounted for.

F ratios are a measure of signal-to-noise and are defined as the ratio between the partial variation explained by the model, relating to each predictor, and the unexplained variation. Larger values indicate a stronger relationship between a predictor and a response, and a value close to unity indicates no statistically significant effect. Activity is determined mostly by T_p and T_p^2 (F ratio 16.6 and 53.2), with the quadratic term reflecting the maxima around 60 °C (Table S10). Other key factors are $T_p c$ and c^2 (F ratio 13.3 and 14.4) which highlight the temperature-concentration coupling that is observed qualitatively in. The quadratic concentration term goes some way to account for the initial decrease in activity before a positive comonomer effect becomes apparent at higher concentrations. Concentration effects are confounded by the change in diffusion regime from the formation of free-flowing polymer particles to soluble gels at higher concentrations which lead to a reduction in activity associated with mass-transport processes.

Table S10 Summary of partial effect tests in model M1: F ratios and p-values for each predictor on each response. Predictors composed of T_p (temperature of polymerisation), *n* (chain length of LAO), and c (*L*AO concentration). $N = 75$.

Predictor	F ratio, *p*-value											
	**Activity**		M_w		**PDI**		T_m		**α**		x	
T_p	16.62	.0001	678.26	<.0001	3.16	.0808	66.60	<.0001	25.51	<.0001	49.52	<.0001
n	1.05	.3105	13.15	.0006	0.54	.4658	5.80	.0193	1.15	.2876	3.42	.0696
c	8.55	.0049	330.29	<.0001	6.59	.0129	86.03	<.0001	136.24	<.0001	139.76	<.0001
T_pn	3.59	.0631	44.90	<.0001	2.31	.1340	4.19	.0453	5.03	.0287	2.48	.1208
T_pc	13.29	.0006	20.01	<.0001	24.23	<.0001	16.47	.0001	1.09	.3020	4.02	.0497
nc	0.19	.6679	5.17	.0267	3.38	.0710	1.35	.2508	14.51	.0003	3.16	.0807
T_p^2	53.17	<.0001	0.69	.4090	0.58	.8111	5.19	.0264	0.23	.6304	6.29	.0150
n^2	6.29	.0150	0.11	.7457	0.11	.7430	0.09	.7717	6.49	.0135	2.16	.1474
c^2	14.41	.0004	26.99	<.0001	0.65	.4232	0.02	.8864	1.25	.2677	0.76	.3877

The cross term T_pc (24.2) is the principal controlling predictor for PDI, which also has moderate contributions to both M_w (20.0) and T_m (16.5). The anticipated dependency of M_w on both T_p and c is reflected in large F ratios for these predictors, 678.3 and 330.3 respectively. Interestingly, of the cross terms, T_pn (44.9) had the largest F ratio, showing that temperature-chain length coupling is a more important factor than concentration-chain length. This is suggestive of a mechanistic interpretation, with larger energy barriers associated with larger monomers interacting with the thermal energy in the system; since polymer molecular weight is ultimately governed by the ratio of chain propagation to termination rate, such a phenomenon is to be expected.

Incorporation is determined almost linearly by concentration, with c being the dominant predictor, alongside a contribution from T_p. The thermal properties of the polymer – T_m and α – depend largely on T_p and c, and therefore secondarily on x. The regression analysis reveals that while T_m is determined principally by the temperature-concentration couple, crystallinity depends more strongly on the side chain length, with the predictors nc, n^2 and T_pn all having statistically significant contributions. This is consistent with physical expectations: increased comonomer concentration (and therefore, incorporation) increases the degree of branching, which reduces the intermolecular forces between polymer chains and lowers the melting point.[10] The branches are generally excluded from the crystalline lamellae, disrupt chain folding and lead to defective crystallisation,[11] with the length of the side chain controlling crystallinity.[12]

Through this analysis, it is possible to quantify many of the features of this system that are observed qualitatively in this work. That the relatively simple regression model M1 captures so much of the chemical and physical behaviour of this highly non-trivial reaction system demonstrates not only the power of large datasets for the delineation of interrelated variables, but also the potential ability to leverage the tuneability of the PHENI* catalyst system towards parameter-space optimisation.

Full details of the model and analysis of variance (ANOVA) are available from the authors upon reasonable request.

12. Designer LLDPE

Table S11 Target, predicted, and measured parameters for the polymerisations P1–3. M1 shown with a ± 95% confidence interval, experimental data shown as mean ± standard deviation.

		Activity /kg_{LLDPE} mol^{-1} h^{-1} bar^{-1}	T_m /°C	α (%)	M_w /kDa	PDI	*x* (wt%)	Conditions	
	Target	*Maximise*	*110*	*Maximise*	*1000*	*Minimise*	-	T_p /°C	61
**P1**	M1	3424 ± 293	112 ± 4	39 ± 4	846 ± 101	4.1 ± 0.4	7.8 ± 1.7	LAO (C*n*)	8
	Experimental	*3175 ± 271*	*112 ± 3*	*39 ± 7*	*606 ± 281*	*4.1 ± 0.4*	*37.8 ± 4.7*	[LAO] /mM	59
	Target	*Maximise*	*100*	*45*	*500*	*5*	-	T_p /°C	80
**P2**	M1	2941 ± 322	107 ± 4	35 ± 4	493 ± 112	4.7 ± 0.4	7.1 ± 1.9	LAO (C*n*)	6
	Experimental	*2552 ± 112*	*117 ± 1*	*44 ± 9*	*153 ± 7*	*3.9 ± 0.1*	*7.3 ± 0.8*	[LAO] /mM	47
	Target	*Maximise*	*80*	*25*	*200*	*6.5*	-	T_p /°C	32
**P3**	M1	3188 ± 1074	95 ± 14	25 ± 13	146 ± 372	5.2 ± 1.3	18.3 ± 6.3	LAO (C*n*)	6
	Experimental	*2286 ± 99*	*58 ± 2*	*23 ± 2*	*355 ± 35*	*3.0 ± 0.1*	*27.5 ± 3.3*	[LAO] /mM	168

Table S12 Points specifying desirability functions for the optimisation of model M1 towards the properties of P1–3.

	Activity /kg_{LLDPE} mol^{-1} h^{-1} bar^{-1}		T_m /°C		α (%)		M_w /kDa		PDI	
	Value	Weight	Value	Weight	Value	Weight	Value	Weight	Value	Weight
**P1**	5000	0.9	120	0.1	75	0.98	1500	0.01	8	0.1
	2000	0.3	110	1	50	0.5	800	1	5.5	0.2
	500	0.05	100	0.1	30	0.05	650	0.01	2.5	0.9
**P2**	5000	0.9	110	0.1	60	0.1	600	0.1	6	0.1
	2000	0.3	100	1	45	1	500	1	5	1
	500	0.05	90	0.1	30	0.1	400	0.1	4	0.1
**P3**	5000	0.9	90	0.1	35	0.1	250	0.1	7.5	0.1
	2000	0.3	80	1	25	1	200	1	6.5	1
	500	0.05	70	0.1	15	0.1	150	0.1	5.5	0.1

In addition to the statistical insight afforded by this highly multidimensional analysis, the real power of statistical models is predictive. Given the immense tuneability afforded by the PHENI* catalyst platform in olefinic copolymerisations, it would be highly desirable to be able to exploit control over the reaction conditions to synthesise polymers with predefined properties. To explore this, three sets of LLDPE properties were devised and translated into desirability functions corresponding to M1, consisting of unimodal functions maximised at the desired value (Figure S32).

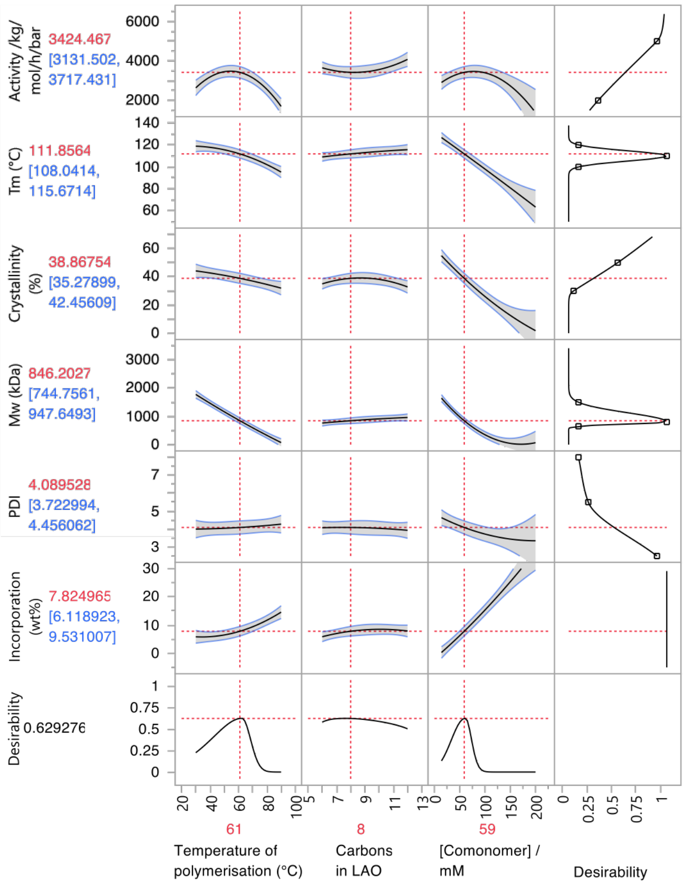

Figure S32 Prediction profiler plots with desirability functions optimised for sample P1: T_m = 110 °C, M_w = 1 MDa, maximised activity and crystallinity, and minimised PDI.

Following multivariate optimisation of total desirability, the calculated values for T_p, Cn, and [LAO] were rounded to an experimentally relevant degree. The expected values of the predicted output properties were found with a 95% confidence interval based on the covariance matrices. These copolymerisations were then performed with otherwise identical conditions to those used throughout this work: 50 mL hexanes, 150 mg TIBA, 2 bar ethylene, and 30 minutes (Figure S33).

Sample P1 was optimised to a relatively unconstrained high M_w (1 MDa) high T_m (110 °C) LLDPE copolymer, the likes of which have already been synthesised within this work. Additionally, the activity and crystallinity were maximised and the PDI minimised. The model M1 converged on a set of conditions (T_p = 61 °C, n = 8, c = 55.5 mM) consistent with the optimised properties and with relatively narrow 95% confidence intervals (T_m = 112 °C; M_w = 846 kDa). Experimentally, the copolymer melting point and molecular weight (112 ± 3 °C; 606 ± 281 kDa) showed a close qualitative fit with the predictions of M1. The relatively large standard deviation in experimental M_w results from the reaction

being at the boundary of the insoluble and soluble regimes, with poor reproducibility between runs. This morphological variation has been noted, and is not explicitly included in M1. Furthermore, the predictions for activity (M1 3424 kg_{LLDPE} mol_{Ti}^{-1} h^{-1} bar^{-1}; expt. 3175 ± 271 kg_{LLDPE} mol_{Ti}^{-1} h^{-1} bar^{-1}), crystallinity (M1 39 %; expt. 40 ± 7 %), and PDI (M1 4.1; expt. 4.1 ± 0.4) were also closely aligned with M1.

Incorporation, *x*, (M1 7.8 %; expt. 37.8 ± 4.7 %) showed a large discrepancy between the prediction of M1 and the experimental result. This is surprising both because incorporation as a function of T_p, *n*, and *c* is one of the best-defined aspects of M1 and because *x* determines many of the other well-predicted properties such as T_m and α. This may in part be due to the borderline solubility of LLDPE of this composition impacting the interpolation. The generally excellent agreement between the desired, predicted, and experimental values highlights the power and utility of a large dataset and statistical modelling in the production of polymers with designer properties.

Samples P2 and P3 were optimised in a more constrained fashion with four variables determined simultaneously. P2 (M_w = 500 kDa; PDI = 5.0; T_m = 100 °C; α = 45%) was interpolated within the scope of previously synthesised copolymers. For P3 (M_w = 200 kDa; PDI = 6.5; T_m = 80 °C; α = 25%), the combination of high incorporation – required for the low crystallinity and melting point – and large PDI is unlike any E/LAO copolymer previously synthesised within this work. As before, in both cases, activity was maximised, and incorporation was unconstrained. Simultaneous quadruplex optimisation resulted in a poorer match between the desired properties and those predicted by the optimised reaction conditions. In particular, the extrapolated properties desired for P3 resulted in poor agreement and large 95% confidence intervals for the predicted values.

P2 was synthesised at T_p = 80 °C, with [C6] = 47.0 mM. The experimental melting point (116.5 ± 0.3 °C) is both larger than the desired value and that predicted by M1. The molecular weight (153 ± 7 kDa) is much less than the desired and predicted values. Otherwise, there is good agreement between M1 and experimentation. The generally good agreement between the predicted and experimental data further demonstrates the power of M1, but the poorer match with the desired properties compared to P1 shows the limits of this methodology for simultaneous optimisation of many intercorrelated parameters.

P3 was synthesised at T_p = 32 °C, with [C6] = 168 mM. The desirability functions were poorly optimised, with the desired values of T_m and PDI (80 °C; 6.5) falling outside the 95% confidence interval of the predicted values of T_m and PDI (95 °C; 5.1). This reflects the lack of data for copolymers having both high incorporation and a high PDI, and perhaps suggests that **1**/TIBA cannot produce such polymers under these conditions. Polymer melting temperature and dispersity (58 ± 2 °C; 3.0 ± 0.1) were substantially lower than both the target and predicted value, and M_w (355 ± 35 kDa) was found to be increased compared with the desired and predicted values. Only crystallinity (23 ± 2%) was well optimised and predicted. In addition to the relatively poor desirability optimisation and low-confidence predictions, this clearly demonstrates the limitations of extrapolating to property-space much beyond the data used to construct the model.

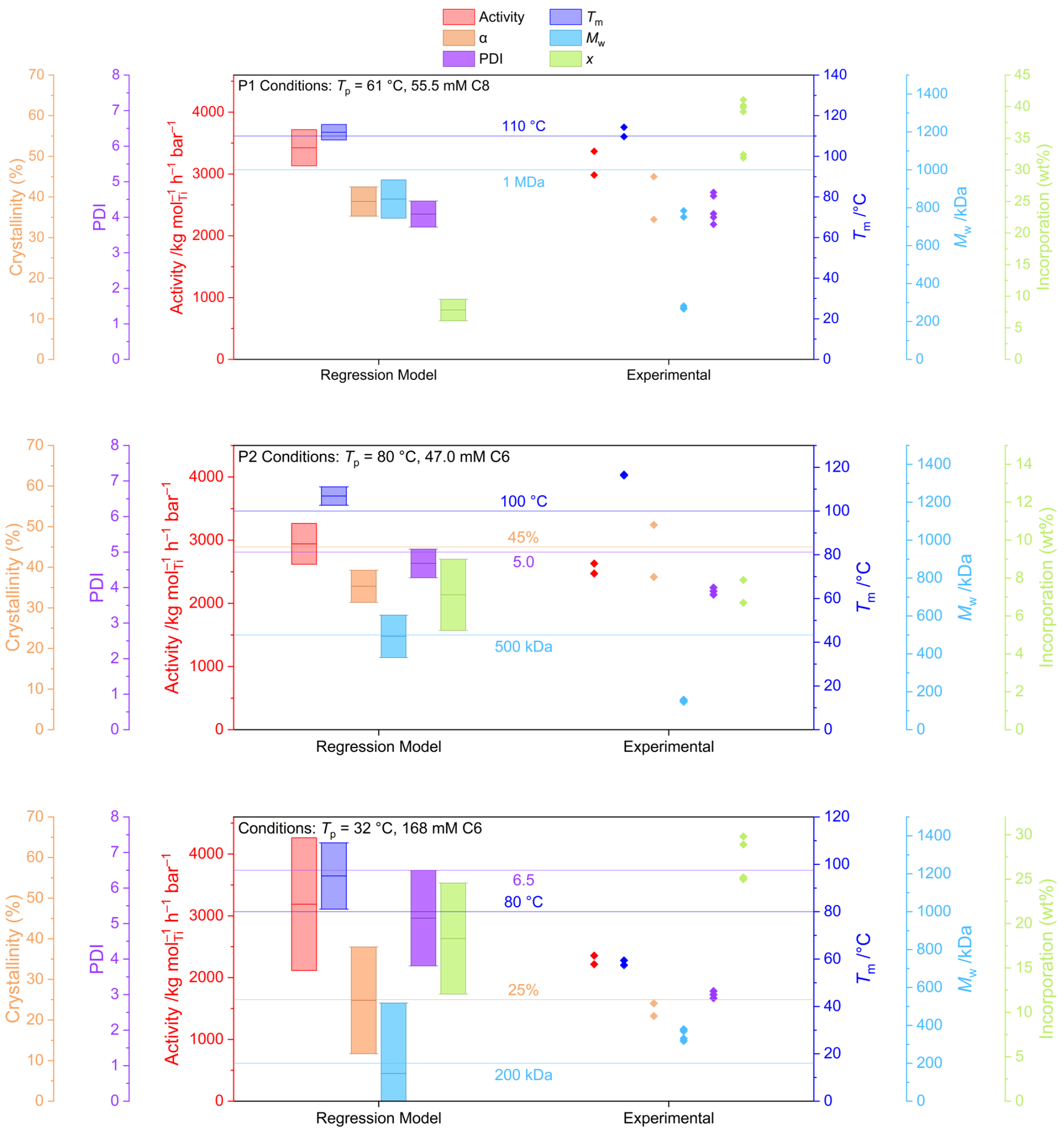

Figure S33 Polymerisation parameters of P1–P3 predicted by the regression model M1 (95% confidence intervals shown) and experimental results. Horizontal lines indicated the target values which determined the optimisation functions for calculating experimental conditions. Polymerisation conditions: 50 mL hexanes, 150 mg TIBA, 2 bar ethylene, and 30 minutes.

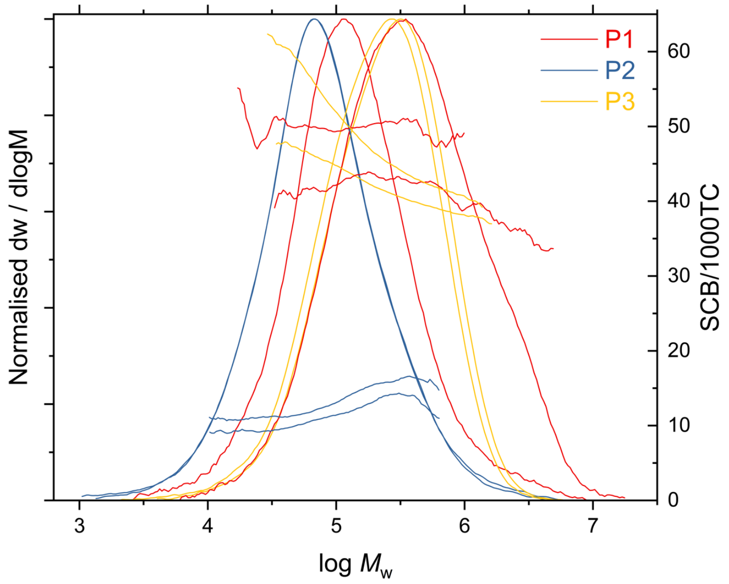

Figure S34 Gel permeation chromatograms of LLDPE samples P1–3 synthesised by **1**. Polymerisation conditions: 10 mg catalyst, 150 mg TIBA, 2 bar ethylene, 50 mL hexanes, and 30 minutes; P1: 61 °C, 463 µL 1-octene; P2: 80 °C, 294 µL 1-hexene; P3: 32 °C, 1050 µL 1-hexene.

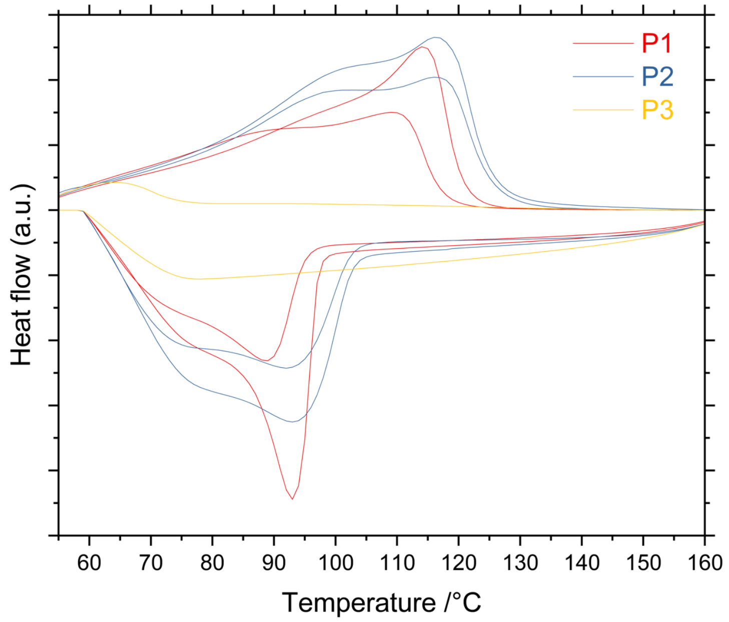

Figure S35 Differential scanning calorimetry of LLDPE samples P1–3 synthesised by **1**. Polymerisation conditions: 10 mg catalyst, 150 mg TIBA, 2 bar ethylene, 50 mL hexanes, and 30 minutes; P1: 61 °C, 463 µL 1-octene; P2: 80 °C, 294 µL 1-hexene; P3: 32 °C, 1050 µL 1-hexene.

Despite the obvious limitations, the potential of synthesising designer polyolefins using a single catalyst on the basis of a statistical model has been demonstrated in principle, and is of potentially enormous industrial significance. Parallelised high-throughput reaction platforms would enable more time- and resource-efficient dataset collection and including additional experimental parameters such as pressure would allow finer control and increase the accuracy of simultaneous multiplex optimisations. Incorporating mechanical and material characterisation into the modelling would further enable a dramatically expanded scope of tunability and control, with the ultimate goal of entirely application-directed synthesis.

13. References

1 D. F. Shriver and M. A. Drezdon, *The Manipulation of Air-Sensitive Compounds*, 2 edn., Wiley, 1986.

2 F. M. Alías, S. Barlow, J. S. Tudor, D. O'Hare, R. T. Perry, J. M. Nelson, I. Manners, *J. Organomet. Chem.*, 1997, **528**, 47-58.

3 J. Tudor, S. Barlow, B. R. Payne, D. O'Hare, P. Nguyen, C. E. B. Evans, I. Manners, *Organometallics*, 1999, **18**, 2281-2284.

4 C. Görl, E. Betthausen, H. G. Alt, *Polyhedron*, 2016, **118**, 37-51.

5 C. G. Collins Rice, J.-C. Buffet, Z. R. Turner, D. O'Hare, *Chem. Commun.*, 2021, **57**, 8600-8603.

6 H. Hanaoka, T. Hino, H. Souda, K. Yanagi, Y. Oda, A. Imai, *J. Organomet. Chem.*, 2007, **692**, 4059-4066.

7 V. Busico, R. Cipullo, A. Mingione, L. Rongo, *Ind. Eng. Chem. Res.*, 2016, **55**, 2686-2695.

8 A. Kamardin, M. N. B. Derman, A. Rahmat, W. Z. A. W. Muhamad, *AIP Conf. Proc.*, 2018, **2013**, 020002.

9 G. B. Galland, P. Quijada, R. S. Mauler, S. C. de Menezes, *Macromol. Rapid Commun.*, 1996, **17**, 607-613.

10 F. J. Stadler, C. Gabriel, H. Münstedt, *Macromol. Chem. Phys.*, 2007, **208**, 2449-2454.

11 S. Watanabe, N. Sano, I. Noda, Y. Ozaki, *J. Phys. Chem. B*, 2009, **113**, 3385-3394.

12 X. b. Zhang, Z. s. Li, H. Yang, C. C. Sun, *Macromolecules*, 2004, **37**, 7393-7400.
